# Operando Investigation of Zr Doping in NMC811 Cathode for High Energy Density Lithium Ion Batteries

**DOI:** 10.1002/cssc.202401796

**Published:** 2025-01-21

**Authors:** Mattia Colalongo, Basit Ali, Nikita Vostrov, Michal Ronovský, Marta Mirolo, Valentin Vinci, Cesare Atzori, Isaac Martens, Peter Kúš, Andrea Sartori, Lide Yao, Hua Jiang, Tobias Schulli, Jakub Drnec, Timo Kankaanpää, Tanja Kallio

**Affiliations:** ^1^ ESRF - The European Synchrotron Radiation Facility 71 Avenue des Martyrs 38000 Grenoble France; ^2^ Department of Chemistry and Material Science School of Chemical Engineering Aalto University Kemistintie 1 Espoo 02150 Finland; ^3^ Department of Surface and Plasma Science Faculty of Mathematics and Physics Charles University V Holešovičkách 2 Prague 8 18000 Czech Republic; ^4^ Umicore Battery Materials Finland Oy Kokkola 67101 Finland; ^5^ Nanomicroscopy Center Aalto University Espoo, Finland 02150

**Keywords:** NMC811, Zr-Doping, XAS, Synchrotron Radiation, Operando HR-XRD

## Abstract

LiNi0.8Mn0.1Co0.1O2 (NMC811) is one of the most promising cathode materials for high energy density Li‐ion batteries (LiBs). However, NMC811 suffers from capacity fading during electrochemical cycling because of its structure instability at voltages >4.2 V vs Li|Li^+^ due to the known hexagonal H2→H3 phase transition. Zr doping has proven to be effective in enhancing electrochemical performances of the NMC811. In depth investigations are conducted through *operando* x‐ray diffraction (XRD) and *ex situ* x‐ray absorption spectroscopy (XAS) measurements to mechanistically understand the benefits of Zr‐doping in a NMC811 material when doped during the co‐precipitation step. Herein, Zr‐doping in NMC811 reduces the formation of the detrimental H3 phase and mitigates the transition metal dissolution upon cycling.

## Graphical abstract

Zirconium (Zr) is employed as a dopant to enhance the electrochemical performance of Ni‐rich cathode materials in Li‐ion batteries. *Operando* studies reveal the underlying mechanism responsible for the increased capacity observed during charging. The doped material fully reacts even at high C‐rates, whereas the undoped material exhibits a sluggish extra‐phase reaction, which lags and limits its overall capacity.

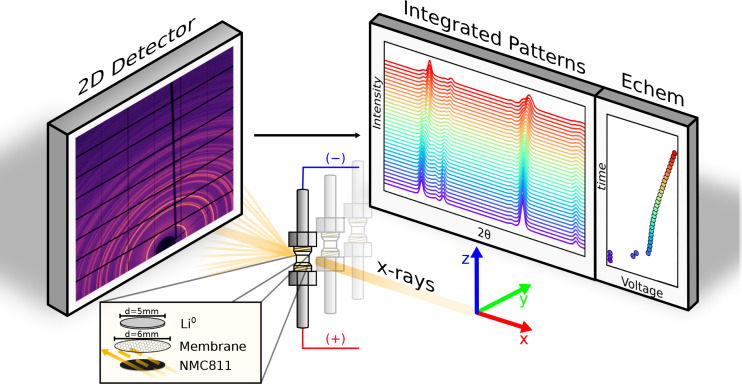

## 1 Introduction

The recent development of the automotive industry toward complete vehicle electrification steeply increased the demand for Li‐ion batteries (LiBs), a rising technology that dominates the global market for electric‐vehicles (EVs) applications.^[1]^ The necessity of high volumetric energy density (Wh/L) LiBs and reduced costs, led the former known LiCoO_2_ cathode material to be overshadowed by Ni‐rich cathode materials. Among the possible Ni‐rich chemistries, an attractive candidate is ${\mathrm{LiNi}}_{0.8}{\mathrm{Mn}}_{0.1}{\mathrm{Co}}_{0.1}O_{2}$ (NMC811), a layered oxide that combines high specific capacity (>200 mAh g^−1^), high thermal stability and low Co and Mn content.[^2^, ^3^, ^4^]

Nevertheless, NMC811 is not reaching its full capability, as it suffers from capacity fading when cycled at high potentials. As many other layered materials, at ~4.2 V, NMC811 undergoes a detrimental phase transformation (between one hexagonal phase to another, H2→H3) which results in an anisotropic lattice collapse along the c‐axis of the unit cell.[^5^, ^6^, ^7^] Furthermore, at deep charge voltages, ${\mathrm{Ni}}^{2+}$ (3b site) migrates in ${\mathrm{Li}}^{+}$ sites (3a site) and vice versa due to their similar ionic radii. This phenomenon is also known as cation mixing, which deleteriously affects the material performance upon cycling.^[8]^

To counter structural instabilities caused by lattice collapse and cation mixing at high voltages, ${\mathrm{Zr}}^{4+}$ bulk doping has been previously proposed as a potential solution for enhancing the cathode cycle life and rate capability.[^9^, ^10^] The benefits of the dopant was mainly attributed to the reduction of cation mixing^[11]^ as Zr−O bonds in the transition metal (TM) site (3b) are stronger than Ni−O, Co−O and Mn−O ones.^[12]^ Additionally, the speculated improvement in specific capacity was linked to the Zr ability to act as a pillar, stabilizing the Li diffusion layer when introduced in the NMC unit‐cell.[^13^, ^14^, ^15^] While cation mixing can be assessed through ex‐situ X‐ray diffraction (XRD) measurements and Rietveld refinement, the pillar effect of Zr remains an open question. The latter phenomenon requires XRD *operando* measurements to investigate the structure evolution while charging. In our previous work, upon co‐precipitation step, we successfully managed to introduce 0.1 mol % vs transition metals concentration of Zr traces in the Ni 3b site of NMC811 material.^[16]^ However, except for the electrochemical data, we are still lack insight into the role of Zr doping mechanism and its effect on the electrochemical improvements reported.

In this study, we provide further understanding on the stabilizing mechanism of Zr in a NMC811 material by investigating it through high‐energy XRD *operando* measurements in Swagelok‐type half‐cells. Based on our XRD measurements, we identify an unreactive phase (phase A) at high voltages in the undoped NMC811 sample, which exhibits slower reactivity compared to Zr‐NMC811. Zr‐doping delays the formation of the most detrimental phase (phase C) at 4.6 V during constant charge voltage (CCV) protocol, revealing Zr role as a structural stabilizer in the NMC811 lattice upon cycling. Phase C is considered the most detrimental phase due to its smaller unit‐cell volume compared to phases B and A. Smaller volume hinders the Li^+^ reintercalation process upon discharge. *Operando* XRD and *ex situ* XAS analysis of the transition metals (TMs) K‐edges show TM dissolution from the NMC811 electrode when aged up to 75 cycles for Zr‐NMC811, supporting the hypothesis that Zr hinders Ni and other TM dissolution during cycling. Transmission electron microscopy (TEM) and phase‐contrast tomography (Nano‐CT) reveal poorer crystalline quality and significant internal porosity in the undoped NMC811 compared to Zr‐NMC811, accounting for the slow‐reacting phase observed at 1 C due to a more tortuous path for Li^+^ ions diffusing from the core to the surface of the secondary particle.

## 2 Results and Discussion

### *2.1. Operando* Structural Variation

**Figure 1:**
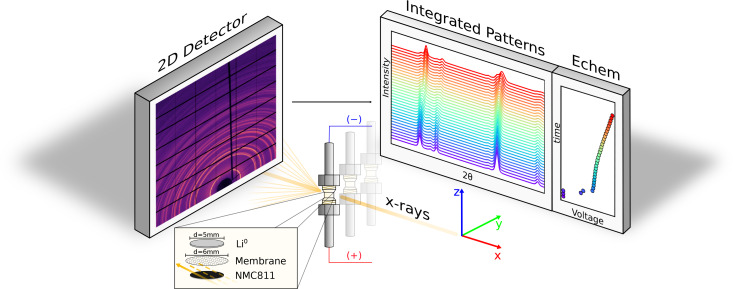
Representative sketch of the *operando* HE‐XRD measurements at the ID31 ESRF beamline. The x‐ray beam of 25×5 μm is focused on the NMC811 positive electrode material. The Swagelok cell is moved along the y‐axis to probe different cathode spots. Each 1 s a 2D snapshot of the relative XRD pattern is collected. The data are later post processed and integrated using the pyFAI python library. The XRD pattern evolution over time is matched with the electrochemistry collected with a Biologic potentiostat.

To effectively probe the average structural variation of the Zr‐NMC811 against the undoped NMC811 sample, *operando* measurements were conducted at high charging voltages. Horizontal movements of the motor that holds the cell allow to raster the electrode with the beam in different spots. A representative sketch of the *operando* set‐up experiment at the ID31 Beamline is reported in Figure 1.

**Figure 2:**
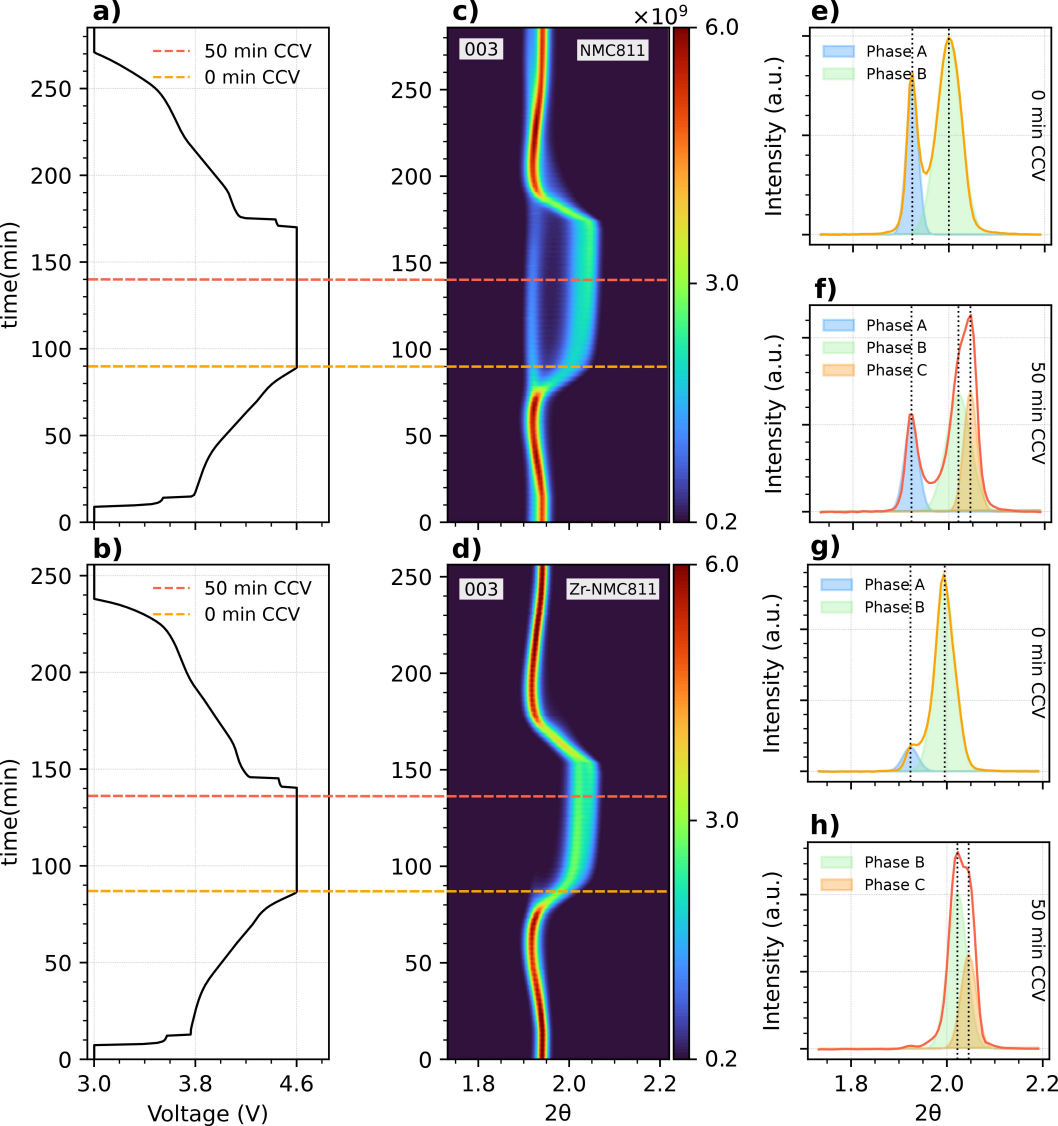
a, b) show voltage vs time profile of undoped and Zr‐doped NMC811, respectively. In c, d) is reported the XRD contour plot of the 003 peak evolution. The orange and red dashed lines in a–d) are referring to 0 min and 50 min after CCV started scan points for the electrochemistry and 2D XRD plot, respectively. e, g) show the 003 peak profile after 0 min of constant charge voltage (CCV). Finally, f, g) represents the 003 peak profile at 50 min of CCV.

For NMC811, the common studied potential window ranges between 3.0 V to 4.4 V vs Li/${\mathrm{Li}}^{+}$ .^[17]^ However, to accelerate the degradation process of the material and be able to detect minor features, undoped NMC811 and Zr‐NMC811 were cycled up to 4.6 V. This upper limit voltage is clearly higher than the H2→H3 (4.2 V), which is stated to be the detrimental NMC811 phase transition.^[18]^ As for layered structures, such as NMC811, the 003 reflection is sensitive to the *c‐axis* evolution upon ${\mathrm{Li}}^{+}$ extraction. At first, the peak shifts to lower 2θ values due to oxygen repulsion between the TM layers resulting in a unit cell expansion. The unit cell expansion from 3.0 V to 4.2 V is known as the H1→H2 solid solution phase transition. Above 4.2 V the 003 reflection undergoes an abrupt sharp variation to higher 2θ values, indicating the typical contraction of the unit cell along the *c‐axis*. The latter variation is known as the H2→H3 phase transition, and the described behaviour matches the typical structural evolution of Ni‐rich cathode materials.^[19]^ A representation of the expansion and collapse of the NMC811 unit cell is reported in Figure S8. In Figure 2 the 003 peak evolution of both undoped NMC811 and Zr‐NMC811 samples is reported. Despite the primary focus of this study being on the 003 reflection due to its sensitivity toward the abrupt contraction of the unit‐cell upon cycling, a broader angle range of the whole XRD pattern is reported in Figure S4. As shown in Figure 2a, b, the electrochemical behavior of the studied samples shows subtle differences in the curve profile. Undoped NMC811 and Zr‐NMC811 were firstly charged at 1 C. Once 4.6 V was reached, a constant charge voltage (CCV) was applied for ~1, to further deplete the cathode of ${\mathrm{Li}}^{+}$ . At the end of the constant current charging cycle (CC), the calculated specific capacity for undoped NMC811 is of 148 mAh g^−1^, whereas for Zr‐NMC811 is of 168 mAh g^−1^. The overall specific capacity of the electrodes at 1 C is lower than that observed in our previous study^[16]^ for the same C‐rate, and also lower than the values obtained from the coin‐cell setup in Figure S3, where the electrodes were cycled at 2 C. However, while Swagelok cells are suited for the scope of this study, they are not the optimal choice for performance evaluations compared to other formats such as coin‐cells. The XRD *operando* data are displayed in Figure 2c, d as a contour plot. Colors from dark blue to red represent the intensity variation, from low to high, respectively. From Figure 2c, the distinct presence of a residual phase is evident and that appears slowly react during the CCV process at 4.6 V. Therefore, the undoped NMC811, above 4.2 V, exhibits clear segregation into two primary contributions: i) a lower 2θ peak, coming from either the H1 or H2 phase and ii) a higher 2θ peak assigned to the H3 phase. Both peaks are found to be present in the sample simultaneously. In fact, over time, H1/H2 decreases in intensity whereas H3 increases accordingly as the slowly reacting H1/H2 phase transforms into H3, indicating a first order phase transition. The presence of two phases at high C‐rates was previously reported in Ni‐rich cathode materials material.^[20]^

To gain a deeper understanding of the phases’ contribution, a contour plot is not sufficient. For a fair comparison between the samples, we extracted the 003 peak profile of the XRD data at two different timestamps. Figure 2e shows the XRD profile of undoped NMC811 in the very beginning of CCV. We are able to identify two phases: in blue the unreacted material (or phase A either H1 or H2) and in green the reacted one (or phase B/H3). Phase A peak is centered at $2\theta =1.924^{\circ }$ and phase B at $2\theta =1.993^{\circ }$ . After 50 min of constant charge, as shown in Figure 2f, the unreacted phase A decreases in intensity, whereas in the reacted H3 peak a third phase contribution (phase C) appears. To avoid confusion, although both phases B and C belong to the reacted H3 phase, hereinafter we will refer to phase B and C as H3 and H3′, respectively, where H3′ refers to a slightly more contracted unit cell compared to H3. At this stage, the three phases are centered at $2\theta =1.924^{\circ }$ , $2\theta =2.022^{\circ }$ and $2\theta =2.044^{\circ }$ for phase A, B and C respectively.

On the other hand, Zr‐NMC811 in Figure 2g shows a barely visible unreacted peak when a voltage of 4.6 V is reached. Consequently, at the upper voltage limit, almost all the powder reacts and transforms into the H3/H3′ phase, in fact, the found phase A amplitude for Zr‐NMC811 is 4 times lower compared to the undoped NMC811 sample. Regarding Zr‐NMC811, as depicted in Figure 2g, the phase A and B are centered at $2\theta =1.930^{\circ }$ and $2\theta =1.997^{\circ }$ respectively, with no substantial difference in the peak positions compared to undoped NMC811. Identically, upon 50 min, although no leftover phase A is present, both phase B and C are clearly detectable from the peak profile. Interestingly, at this timestamp the peak position of the two phases are $2\theta =2.020^{\circ }$ and $2\theta =2.044^{\circ }$ for B and C, respectively, almost identical to the undoped NMC811 sample reported in Figure 2f. The errors on the peak position fit are not reported since they are below 0.06 % and smaller than the angular resolution reported in Figure 2f. The errors on the peak position fit are not reported since they are below 0.06 % and smaller than the angular resolution reported in subsection 4.3. At this stage, it is evident from Figure 2 that the modified Zr‐NMC811 allows an even ${\mathrm{Li}}^{+}$ extraction throughout the cathode material because of the almost complete absence of the Li‐rich H1 (or H2) structure (phase A) upon CCV compared to undoped NMC811.

Each phase (A, B and C) at 4.6 V share the same 2θ value for the undoped NMC811 and the Zr‐NMC811 sample. This does not correlate with the pillar effect hypothesis, where we would expect a lower contraction magnitude of the unit‐cell in the doped sample, especially at high delithiation states. However, detecting the pillar effect might be challenging at such low doping levels, as it could fall below the measurement sensitivity. It is also important to note that the 0.1 mol % Zr concentration relative to the total amount of other transition metals (Ni, Co, Mn) is quite minimal, and we lack evidence on whether ID31 is sufficiently sensitive to detect peak shifts from homogeneous strain at this level.

**Figure 3:**
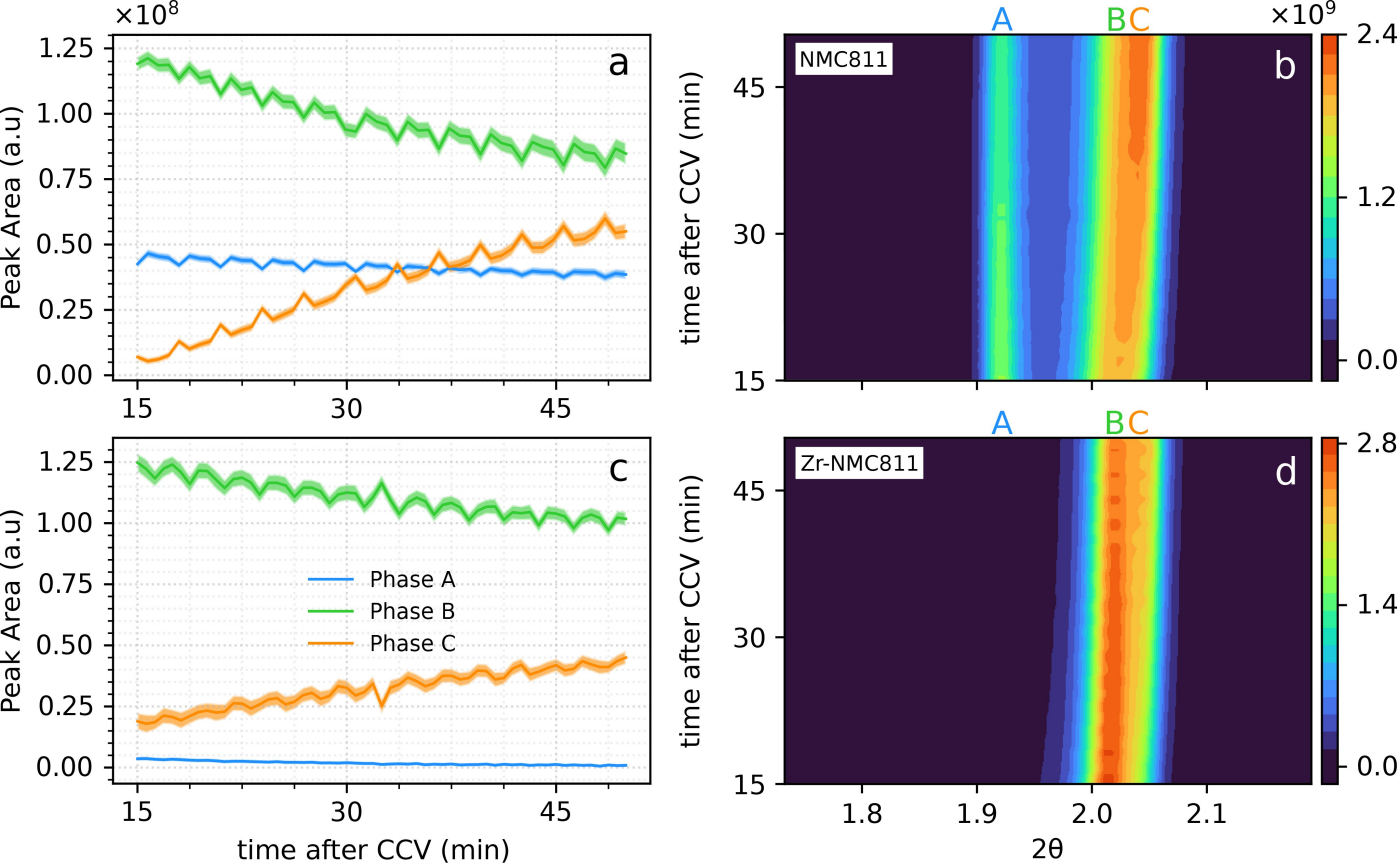
a, c) peak area calculation (amplitude) of both undoped NMC811 and Zr‐NMC811 for the three phases, A, B and C. The observed amplitude oscillation are due to the different amount of cathode material hit by the beam at each Y scan. Possibly due to different cathode porosity at different Y positions. b, d) Contour plot of the undoped NMC811 and Zr‐NMC811 samples magnified in the region from 15 min to 50 min after the CCV (at 4.6 V) mode started. On top of the contour plot the letters A, B and C refers to the homonym phases. The letters are located where the phases are centered at 50 min of CCV.

Curiously, by focusing on the 003 XRD peak in the time window of 15–50 min after starting the CCV mode, multiple phases can be detected, and their corresponding amplitude can be retrieved. As reported in Figure 3a for the undoped NMC811 sample, within the first minutes of holding potential, the phase C is hardly detectable. As shown in Figure 3b, at 15 min after CCV started, the phases B and C are very close to each other. The reacted phase (H3 and H3′) at initial stage of CCV corresponds to a broad single peak, therefore, phase B and C are not yet well‐defined. When the peak profile becomes clearer, the model is capable to recognize phases A, B and C together with a higher accuracy. Notably, both phase B and phase A contribute to the phase C evolution, as they both decrease over time.

However, Zr‐NMC811 reported in Figure 3c, shows the expected low contribution of the phase A that continuously decreases. Moreover, for the reacted H3+H3′ peak on the right in Figure 3d, the phase B and C are easily distinguishable. Evidently, the increase of the phase C over time is slower as compared to the undoped NMC811 sample. Although the formed phases share the same 2θ values, the growth rate of the most detrimental phase (phase C) is slower in Zr‐NMC811. It needs to be underlined that the lattice collapse observed when forming the H3 and H3′ phases, are the main cause of irreversible structural damage,^[21]^ as shown by the curves in Figure 3. Among the phase B and C, the latter phase possesses a deeper structural collapse, which is considered to be more detrimental for the overall cathode stability. A too deep unit cell collapse hinders the ${\mathrm{Li}}^{+}$ reintercalation upon discharge, reducing the capacity of the cathode material. In Figure 3a and 3c, the observed oscillations are attributed to variations in electrode porosity across different Y‐probed spots. This idea is supported by plotting data from only one Y position, as shown in Figure S5, where the oscillations are no longer present. The oscillations recur every four cycles, corresponding to the four Y spots probed on both undoped NMC811 and Zr‐NMC811 electrodes. Therefore, the described behaviour underlines the capability of the ${\mathrm{Zr}}^{4+}$ to act as a pillar when included in the host structure, not fully avoiding the contraction of the unit cell, but possibly slowing down the process at high voltages. Furthermore, it has been reported by Jung et al. that the shrinkage of the c‐parameter at the H2→H3 phase transition for NMC811 is ascribed to a decrease in repulsion between the oxygen layers, caused by O_2_ release.^[22]^ We, therefore, may assume that, because of Zr−O stronger bonds compared to Ni−O, Co−O and Mn−O bonds in NMC811, the evolution of the phase C formation is hindered by a lower oxygen release at 4.6 V for the Zr‐NMC811.

### 2.1 Ex‐Situ X‐ray Absorption Spectroscopy for Oxidation State Evaluation in Aged Electrodes

To understand the role of Zr on the NMC811 lattice, especially its effect on the TMs, x‐ray absorption spectroscopy (XAS) was employed. XAS is a versatile tool that allows to study the variation of elemental oxidation state and changes in local environment of a probed species. The information retrieved by means of XRD are often complemented by exploiting the XAS technique, which gives the full picture of the degradation mechanisms. Further to the structural variation upon charge, XAS measurements were mainly performed to focus on the possible effect of Zr‐doping on the TM oxidation state.

For this reason, Ni, Co and Mn K‐edges ex‐situ spectra were collected on undoped NMC811 and Zr‐NMC811 electrodes. The measurements were performed on fresh electrodes (non‐cycled) and aged electrodes, cycled 75 times at 2 C between 3 V to 4.4 V until consistent degradation was achieved. The cycling data of the aged cells are reported in Figure S3.

**Figure 4:**
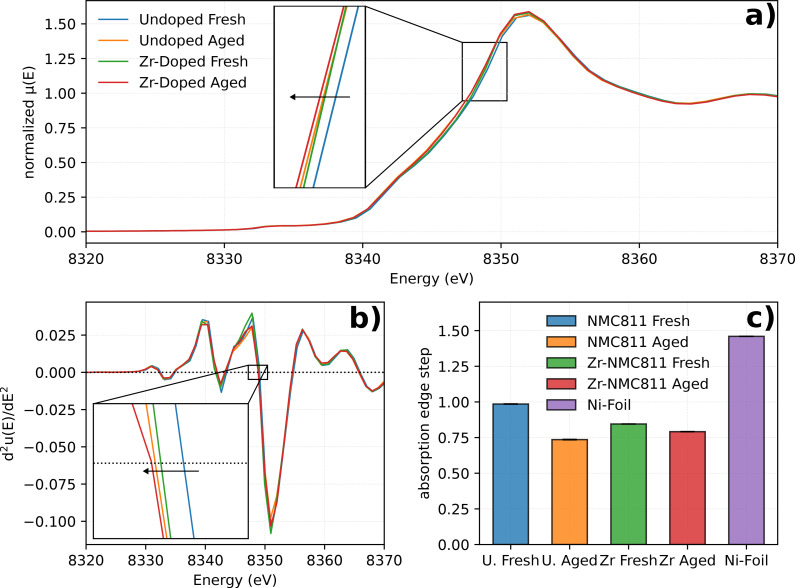
Ni K‐edge of the fresh and aged undoped NMC811 and Zr‐NMC811 samples. In a) XANES portion of the Ni K‐edge and a zoomed inset close to magnify the *E*_0_ region. In b) the second derivative of the absorption signal within the same energy domain. Zoomed in portion in the same plot that shows the intersection with the experiment data at d^2^μ(E)/dE^2^=0. c) Extracted edge‐step values from Athena software. Error bars are barely visible but are calculated by considering the standard deviation over 4 scans values.

As shown in the zoomed inset of Figure 4a, the shift variation of the Ni K‐edge for the measured electrodes indicates a difference in the TM oxidation state. Specifically, the absorption edge of all the samples undergo a lower energy shift compared to the undoped NMC811 fresh sample. Schipper et al. reported that Ni tends to be reduced to lower oxidation states when Zr is present in the host structure.^[12]^ Therefore, ${\mathrm{Ni}}^{2+}/{\mathrm{Ni}}^{3+}$ ratio increases as Ni partially reduces its oxidation state to sustain the charge neutrality upon ${\mathrm{Zr}}^{4+}$ introduction. This explains the shift to lower energies of the Zr‐NMC811 fresh sample compared to the undoped NMC811 fresh sample. However, what remains delicate to understand is the reason of a shift to lower energies of the absorption edge for both aged samples compared to the fresh ones. One hypothesis is linked to the TM dissolution from a Ni‐rich host materials,[^23^, ^24^] which leads to an apparent reduction of Ni oxidation state upon cycling as the local environment of the TM becomes richer in ${\mathrm{Li}}^{+}$ as the Li/Ni ratio increases.[^25^, ^26^] To better visualize the oxidation state variation of nickel, the second derivative of the absorption signal can be exploited. As shown in Figure 4b, the fresh undoped NMC811 intersect 0 at 8349.08 eV, the aged undoped NMC811 sample at 8348.90 eV, the fresh Zr‐NMC811 at 8348.94 eV and the aged Zr‐NMC811 at 8348.88 eV. Clearly, the highest energy shift difference is of 1.04 eV coming from the undoped NMC811 upon cycling. Although Ni is the main redox ion in Ni‐rich cathode materials, features variation are expected to be more prominent on this specific TM.^[27]^ However, surprisingly, for undoped NMC811 also Mn and Co undergo a lower oxidation state shift upon cycling, whereas for the Zr‐NMC811 the oxidation state remains similar to the fresh sample, as reported in Figures S9 and S10. Ni, Mn and Co dissolution was previously reported in Ni‐rich materials due to the continuous loss of oxygen upon high charge voltages.^[28]^ While it is essential to consider the potential impact of these findings, it is equally important to underline that the observed changes are quite subtle. Therefore, any interpretation of the data should be approached with caution, keeping in mind the small amount of these shifts.

A reliable indicator to inspect possible TM dissolution from the positive electrode relies on the examination of the K‐edge absorption jump. Neglecting the auto‐absorption phenomena derived from ~100 ηm thickness electrode, the edge step, to a first approximation, is sensitive to the probed species concentration. As shown in Figure 4c, upon cycling the undoped NMC811 sample undergoes the largest edge absorption variation, confirming the hypothesis of metal dissolution from the cathode material.^[29]^ For data completeness, although no direct comparison with undoped NMC811 is possible, the Zr K‐edge spectrum of Zr‐NMC811 sample, was further measured. As reported in Figure S11, after cycling the local environment of Zr remains unchanged, and no variation of the absorption edge is detected.

A more detailed examination of the TM dissolution process was conducted by analyzing the Celgard membranes, which serve as separators in the coin cells, using x‐ray fluorescence (XRF) measurements at the BM23 beamline. This investigation focused exclusively on the aged cathode samples, where metal dissolution from the cathode is expected to occur. The XRF measurements were performed at an energy of 8529 eV, above the Ni K‐edge, to ensure detection of the three primary transition metals. As shown in Figure S12, the Ni concentration on the Celgard membrane was higher for the undoped NMC811 compared to the Zr‐NMC811. The presence of Ni element onto the separator indicates the cathode material dissolution and the transfer of Ni element from the cathode to the separator through the electrolyte.

Hence, we observe a dual effect of Zr doping in the NMC811 sample. Firstly, it diminishes the rate at which the extra phase C forms during cycling at high voltages. Secondly, it decreases the dissolution of TMs. The decrease in TM dissolution is likely attributed to the Zr capability to diminish the oxygen release, when included into the host structure, due to its stronger Zr−O bonds.^[10]^ At high voltages (4.6 V), when ${\mathrm{Li}}^{+}$ is removed from the cathode structure, the Ni oxidation state shifts from 3+ to 4+. ${\mathrm{Ni}}^{4+}$ species, have a smaller ionic radius compared to 3+ and 2+ oxidation states.^[30]^ However, Ni^4+^‐O bonds are unstable and tend to reduce back to Ni^2+^ state, releasing oxygen in the process.^[31]^ The loss in oxygen and the lower oxidation state of the Ni (and other transition metals) when the material is cycled can be linked, therefore, to a permanent oxygen loss from the structure. Furthermore, the oxygen lost, for LiPF_6_ based electrolytes, tends to react with protic impurities in the electrolyte forming HF, acidic by‐product which attacks the active material leading to the measured metal dissolution.^[32]^ It is possible that the smaller energy shift observed in the absorption edge of Zr‐NMC811 during cycling indicates that the dopant may help reduce oxygen evolution, likely due to the stronger Zr−O bonds compared to those with other transition metals in NMC811. However, in the absence of direct O_2_ evolution data, this interpretation should be taken as a hypothesis, highlighting the need for further investigations related to the gas evolution process.

It is important to highlight that the effects observed through XRD and XAS are rather small, and the reason for the significant presence of the slow‐reacting phase A in the undoped NMC811 sample is still unclear. Therefore, further characterizations are required.

### 2.2 Morphological Characterization Through SEM and TEM

The co‐precipitation method requires stringent control over multiple parameters as the secondary particle morphology can be tailored by temperature, atmosphere, pH, chemical composition, reaction time, and stirring rate. Any small variation of one of the mentioned parameters can lead to a large difference in particle morphology, size, and tap density,^[33]^ especially when the TM ratio is modified.[^34^, ^35^] However, the precise synthetic‐route of the co‐precipitation step for the samples analysed here cannot be disclosed, which is a common academic challenge when precursors from industry are used.

**Figure 5:**
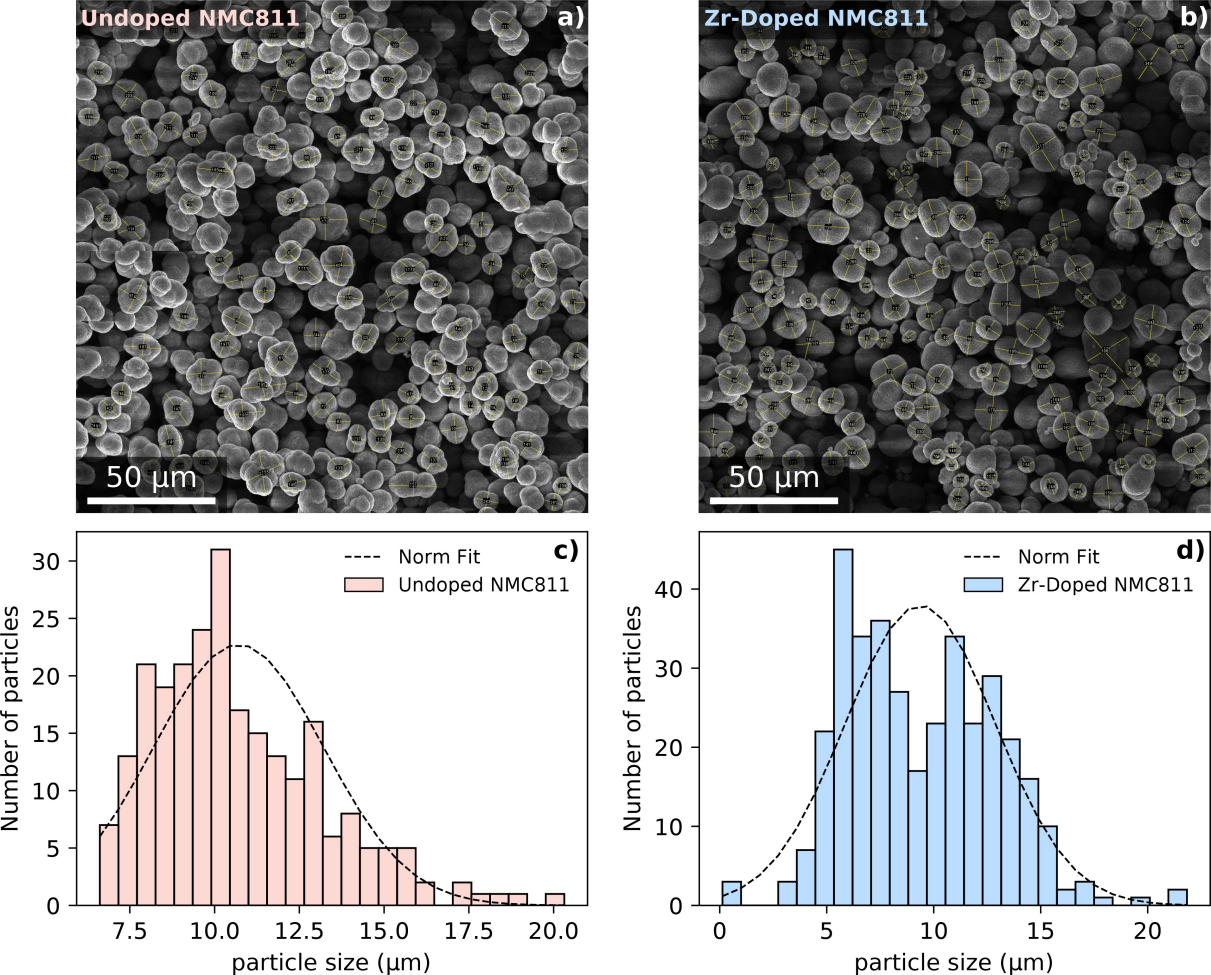
In a, b) SEM images of the undoped NMC811 and Zr‐NMC811 powders after synthesis. As the particles have not an exact round shape, particle size is determined by crossing over 120 particles. In c, d) the particle distribution size of both doped and undoped NMC811 is reported.

Figures 5a and 5b show the secondary particle distribution of the undoped NMC811 and Zr‐NMC811 powder right after synthesis, respectively. Because of the elliptical shape of the particles, two axis (major and minor) were considered within each particle, leading to a statistics of ~120 secondary particles. At a glance, it is clear how both samples possess a different particle distribution. The Zr‐NMC811 sample shows a bimodal distribution. By fitting the histogram with a normal distribution function as shown in Figures 5c and 5d, undoped NMC811 is shown to possess an average secondary particle size of (10.7±2.5) μm and Zr‐NMC811 of (9.3±3.5) μm. Although the fit weighs the totality of the histogram, it can be seen in Figure 5d the presence, for Zr‐NMC811, of a second ensemble of particle distribution that centers at around 6–7 μm which results in lowering the 11 μm average particle size. Lipson et al. suggested that when a dopant is added to the TM solution upon precipitation, it generates additional nuclei in the reactor reducing the average particle size distribution and altering the particle morphology, even when the other synthesis parameters are unchanged.^[36]^ The changes observed in the lithiated samples through SEM analysis may thus originate from the initial precipitation stage, where zirconium (Zr) is added as an additional salt to the reactor.

**Figure 6:**
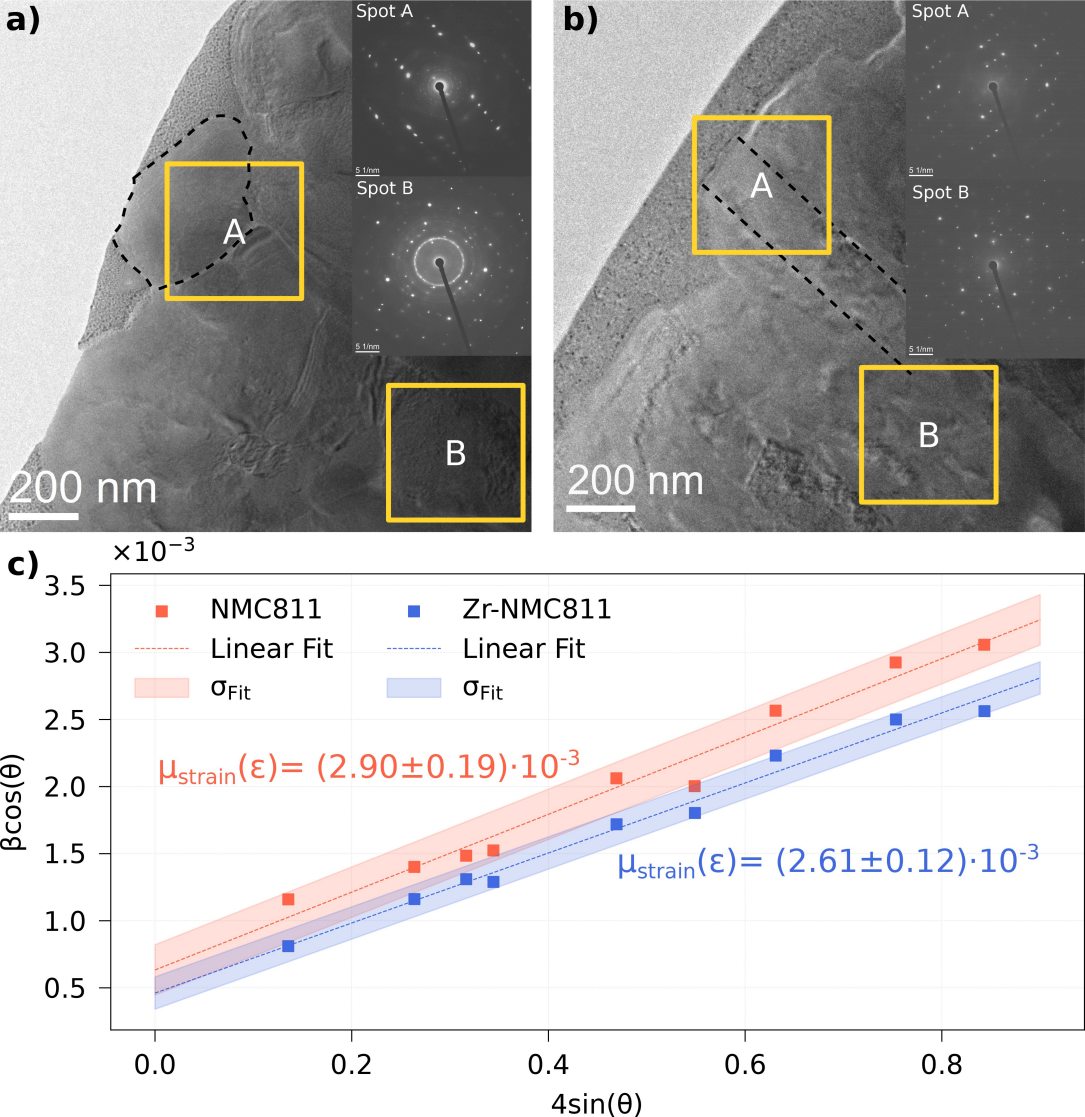
a) undoped NMC811 TEM lamella. In yellow squares, the selected area electron diffraction (SAED). A spot is close to the surface and B is deeper to the bulk. SAED patterns are displayed within the TEM lamella picture. b) Zr‐NMC811 TEM lamella. Spot A and B in yellow squares represent the SAED area. SAED patterns of A and B spots are reported within the TEM lamella picture. c) Williamson–Hall (WH) plot of both undoped NMC811 and Zr‐NMC811.

To further investigate inner secondary particle morphology, ~100 nm thick TEM lamellas of undoped NMC811 and Zr‐NMC811 were studied. In Figure 6a, the TEM lamella of the undoped NMC811 sample is reported. Particular attention needs to be pointed at the selected area electron diffraction (SAED) of the A and B spots. In fact, the bulk spot B shows a spotty diffraction pattern but also ring‐like features for multiple reflections. The same is observed for the spot A, which is closer outermost particle layer. Although the diffraction rings are less evident, those are still present, but closer to the beam stop for undoped NMC811. In contrast, SAED images in Figure 6b, ascribed to the Zr‐NMC811 sample, do not show any diffraction ring in neither spot A nor B (surface and bulk). Their absence for Zr‐NMC811 suggests less misoriented crystal grains, which, within the probed spot, indicates better crystalline ortientation..[^37^, ^38^, ^39^]

Focusing on Figure 6a, highlighted with a black dashed line, the visible crystals show more equiaxed morphology compared to the Zr‐NMC811 reported in Figure 6b, where the primary particle grains elongate from the surface toward the bulk extending radially.

This type of morphology change upon doping was previously reported by Park et al.^[40]^ A radial distribution of primary particles defines less tortuosity for ${\mathrm{Li}}^{+}$ diffusion among crystal grains.[^40^, ^41^] In this specific case, the reason behind the inactivity of the phase A, shown in Figure 3, can be linked to an apparent difference in morphology of the primary particles which slows down the ${\mathrm{Li}}^{+}$ diffusion path among the grains distributed in the secondary particles. However, the statistical limitations dictated by the TEM lamellas and SAED spot size may raise doubts about the representativeness of the previous assumption translated to sample average. To further assess the crystal quality of the two samples, better statistics can be reached by analyzing the Full Width at Half Maximum (FWHM) evolution vs 2θ of the whole XRD for both undoped NMC811 and Zr‐NMC811. The FWHM is calculated on the open circuit voltage (OCV) XRD patterns before the charging process begins. Nine, well separated, single reflections were selected in the respective XRD patterns. Precisely the 003, 101, 104, 10$\bar{5}$ , 113, 20$\bar{4}$ , 208, 217 and 21$\bar{11}$ located at 1.322 Å^−1^, 2.573 Å^−1^, 3.089 Å^−1^, 3.360 Å^−1^, 4.584 Å^−1^, 5.366 Å^−1^, 6.175 Å^−1^, 7.380 Å^−1^ and 8.272 Å^−1^ in q‐space, respectively. As displayed in Figure S7, the FWHM variation vs the scattering angle is overall higher for the undoped NMC811 sample. However, assuming a constant instrumental broadening, the peak width (FWHM) depends on two main factors, crystal size and microstrain ($\mu_{\mathrm{strain}}$ ). The latter is proportional to defects, lattice distortions and grain boundaries concentration, having no mechanical stress applied from external sources or thermal effects. To separate the effect of strain and size in each sample, the Williamson–Hall (W−H) analysis can be used to differentiate the two contributions separately.^[42]^ In our case study, the values of $\mu_{\mathrm{strain}}$ (or slope) have been extracted from the W−H plot as reported in Figure 6c. The $\mu_{\mathrm{strain}}$ value is lower for Zr‐NMC811 compared to the undoped NMC811 one. This result suggests that the Zr‐NMC811 sample possesses overall higher crystal quality, possibly due to reduced lattice distortions as seen from data collected in the SAED‐TEM measurements and reported in Figure 6a, b.

### 2.3 Secondary Particles Porosity Phase‐Contrast X‐ray Tomography

As mentioned previously, the main limitation of studying TEM lamella is related to the realistic representativeness of the examined volume. Specifically, we are constrained by a 100 nm thick lamella where the SAED spot size or field of view (FOV) can be as small as a few hundred nanometers.^[43]^ To obtain more representative investigation of the inner primary/secondary particle morphology, it would be beneficial to use FIB milling by cutting an entire secondary particle and examining the structural features of the resulting cross‐section. However, particle milling is rather challenging, time‐consuming and destructive, limiting FIB‐SEM cross‐section images to be non statistically representative since usually limited to one cross‐section. Despite lower resolution compared to FIB‐SEM, the realistic advantage of Nano‐CT (computed tomography), for secondary particle morphology, is mainly ascribed to the ability of exploring non‐destructively multiple particles within a chosen 3D volume.

**Figure 7:**
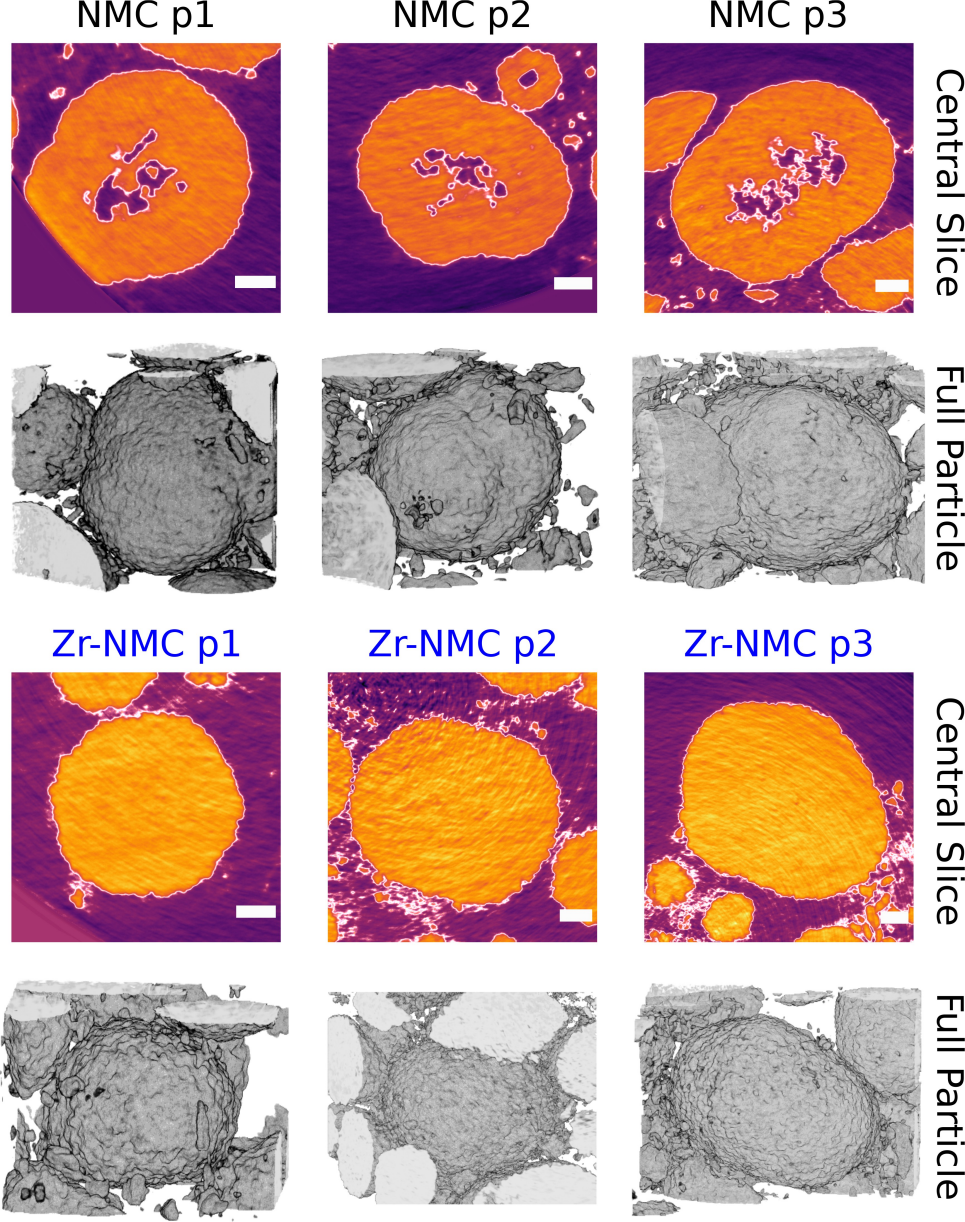
Nano‐CT measurements of three different undoped NMC811 and Zr‐NMC811 secondary particles. The 1st and 3rd rows show the 2D cross section slice inside the particles core for undoped NMC811 and Zr‐NMC811 respectively. The 2nd and 4th rows show the 3D rendering of the outer shape of the selected particles. In the 1st and 2nd rows, the scale bar size is 2 μm.

As Zr‐NMC811 shows a smaller average secondary particle size distribution, the investigation of internal particle morphology was conducted mainly on equivalent particle size between undoped NMC811 and Zr‐NMC811. As shown in Figure 7, three particles for each sampled volume were chosen. The middle internal cross‐section structure is reported in Figure 7 1st and 3rd rows. Interestingly, undoped NMC811 secondary particles possess an evident internal porosity, whereas the same cannot be individuated for the Zr‐NMC811 sample. By paying attention to the 2nd row of Figure 7 where the 3D reconstruction of the outer shell of the particles is shown, all the large segmented particles resemble those agglomerates seen in Figure 5. The undoped NMC811 particles’ porosity is not randomly dispersed through the particle, but it mainly clusters in the center of the middle slices, as reported in Figure S14. Notably, the highest concentration of particle′s voids are also highly localized in the particle centre, and not close to the surface. On the other hand, the Zr‐NMC811 secondary particles do not present any porosity regardless the cross‐section as reported in Figure S15. Voids almost certainly have a negative impact on ${\mathrm{Li}}^{+}$ diffusion during charge and discharge, disrupting Li‐ion conduction^[44]^ pathways between the particle core and surface. Moreover, Li‐ion disrupted pathways become more important at fast charge rates such as 1 C. For this reason, the presence of internal voids (indicative of greater crystal disorder), resulted in higher μ_strain_ and inferior crystal quality for undoped NMC811, which ultimately led to the formation of the unreacted phase A, a phase that shows, kinetically, slower ${\mathrm{Li}}^{+}$ extraction upon charge from the cathode material.

## 3 Conclusions

A comprehensive study of Zr bulk doping in a NMC811 sample was performed to better understand the kinetics and degradation mechanism of the undoped sample and the dopant efficacy. *Operando* XRD measurements were conducted to study the effect of the dopant on the NMC811 structural evolution upon charging. The presence of 3 phases (phase A, B and C) were detected at high charging voltages (>4.2 V) for the cathode material. The slow reactive phase A, was mainly found in the undoped NMC811 sample, limiting the extraction of ${\mathrm{Li}}^{+}$ from the layered material. On the contrary, the almost complete conversion of the phase A allowed Zr‐NMC811 to fully react at the upper voltage of 4.6 V. No clear peak position variation between Zr‐NMC811 and undoped NMC811 was found at 0 min and 50 min after the CCV process started. However, the higher transformation rate to the most detrimental phase (phase C) at constant voltage of 4.6 V, was observed to occur in the undoped NMC811 sample. Furthermore, XAS studies have shown that Zr substitution can potentially help the whole NMC811 system to reduce the dissolution of its most abundant metal, as for undoped NMC811 sample a higher concentration of Ni is found on the Celgard membrane, indicating transition metal dissolution from the cathode to the separator. Moreover, we observed that primary and secondary particle morphology changed upon doping during the co‐precipitation method.

Although further studies are required, the greater unit cell contraction observed in undoped NMC811 suggests that Zr may act as a stabilizing element within the unit cell. While it does not, completely, prevent the formation of the most detrimental phase (phase C), it appears to slow down its development. One interpretation to metal dissolution and delayed structural collapse for the Zr‐NMC811 sample, can be ascribed to the stronger Zr−O bonds formed in the ${\mathrm{TMO}}_{6}$ octahedral framework. The stronger TM−O bonds can provide mechanical support to the structure, preventing structural collapse in the absence of ${\mathrm{Li}}^{+}$ at high charging voltages, and mitigating oxygen loss during cycling. However, to correctly address these hypotheses, further investigations could focus on the gas evolution of these samples upon cycling. It is worth considering that the morphology difference and crystalline quality found in Zr‐NMC811 can be linked to a more uniform ${\mathrm{Li}}^{+}$ extraction across the cathode material at high C‐rates compared to undoped NMC811, due to the absence of internal porosity for the Zr‐NMC811 secondary particles. This study reinforces the importance of doping, particularly during co‐precipitation, for the development of high‐energy density Ni‐rich cathode materials for Li‐ion batteries.

## Experimental

### Material Synthesis

The precursor materials NMC(OH)_2_ and the doped NMCZr (OH)_2_, as received from Umicore Battery Materials Finland, have been synthesized by a co‐precipitation route. The precursors are mixed with LiOH (Thermo Fisher, Anhydrous, 98 %) in a 1 : 1.005 ratio in a mortar and gently ground together with a pestle for 20 minutes to obtain a homogeneous distribution between the powders. The 0.5 % excess of Li is added to tackle the Li evaporation upon solid state synthesis. The mixture is calcinated at 800 °C under O_2_ atmosphere with a flow of about 100 ml min^−1^ for 12 h in an alumina boat to obtain undoped NMC811 and Zr‐NMC811 samples. Zirconium concentration and its presence as a bulk dopant is discussed in our previous study.^[16]^ NMC811 electrode preparation consists of 93 wt % of active material (undoped NMC811 and Zr‐NMC811), 3 wt% conductive carbon (Timcal Super C65), and 4 wt% polyvinylidene fluoride (Solvay, Solef 5130) in N‐methyl‐2‐pyrrolidone (NMP from BASF, Life Science). The slurry was cast on an Al foil (MTI Corporation, 18 μm thickness) with a doctor blade and an electrode thickness of ~120 μm. The paste was dried overnight in the fume hood and then heated for 4 h at 80 °C into an oven to remove the last NMP residuals.

### Cell Preparation

A commercial, 1/4′′ diameter Swagelok‐type (S4R, France) cell was used for the diffraction experiment. The commercial PTA body that comes from the supplier was replaced with a polyether ether ketone (PEEK) body due to its low x‐ray absorption to x‐rays and unreactivity with both metal Li and the ${\mathrm{LiPF}}_{6}$ electrolyte.^[45]^ The half‐cell was prepared by cutting a cathode of 5 mm in diameter, a 1/4′′ (~6.25 mm) Celgard separator and 5 mm diameter Li foil (99.9 % purity, 0.75 mm thickness, VWR), used as a counter‐electrode. 20 μL of 1.0 M ${\mathrm{LiPF}}_{6}$ in ethylene carbonate/ethyl methyl carbonate (EC : DMC) 1 : 1 (Sigma–Aldrich, LP30) was added as an electrolyte. The SP‐300 Biologic potentiostat was used to control the cell potential and current. Each electrode in the Swagelok cell underwent a formation cycle at 0.025 C (see Figure S2) and then cycled at 1 C in the voltage window 3 V to 4.6 V. At the upper voltage limit, a constant voltage is applied to continue the lithium extraction for ~1 h. The current applied at 0.025 C is calculated considering an NMC811 theoretical capacity of 190 mAh g^−1^ and their respective active mass of the electrode, whereas the current applied at 1 C is calculated from the formation time each cell took upon charge and discharge.

### XRD Data Collection

*Operando* x‐ray diffraction (XRD) was collected at ESRF beamline ID31 using an X‐ray wavelength of 0.161 Å (77 keV). The beam size of 25×5 μm^2^ (horizontal x vertical) was used to illuminate the cathode in “grazing incidence” mode, where the beam is parallel to the plane of the electrode. The data was collected using a Pilatus 2 M CdTe detector exposing the sample for 1 s per pattern. The sample distance and data integration was retrieved by using the PyFAI package^[46]^ and the CeO_2_ NIST SRM 674b standard. The CeO_2_ fit through the pyFAI package estimated an angular resolution of $∼0.003^{\circ }$ for the beamline setup and sample‐detector distance. In order to mitigate the influence of the beam on the NMC electrodes in the *operando* cell, attenuators were used to reduce the beam flux. Prior to the sample, the photon flux was of ~7.65×10^9^ photons s^−1^. The beam dose per scanned point in the cell is estimated to be 38.4 kGy, significantly lower than the critical threshold value identified by Jousseaume et al. at which beam‐induced damage effects begins to manifest.^[47]^ The calculations for beam dose were conducted using the open Jupyter Notebook tool accessible at https://github.com/STardif/dose_estimation. To further minimize the dose at a single probed location, the cell was shifted along the stage′s y‐axis (see Figure 1). In total, four different electrode spots were selected alternately. As soon as the XRD pattern snapshot was taken, the motor moved to a different electrode spot where another pattern was, next, collected. Considering 1 s for an XRD 2D snapshot and 3 s for the stage motor to move from one electrode spot to the next one, between each XRD, ~4 s have passed. Therefore, the voltage resolution upon charge of each XRD pattern between 3.5 V–4.6 V at 1 C is of 1.2 mV/s^−1^.

### XRD Data Analysis

To identify multiple phase appearance, especially at high voltages, two pseudo‐voigt functions from the *lmfit* python module^[48]^ were used. The initial guess position of the peaks was identified through the 2^*nd*^ derivative local minima of the diffraction peak as shown in Figure S6. More details are reported in Supporting Information,.

### Phase‐Contrast Tomography (Nano‐CT)

To gain insights into the morphology of the cathode active material secondary particles, phase‐contrast tomography was used. Tomographic scans were conducted on the undoped NMC811 and Zr‐NMC811 fresh and aged electrodes at the ID16B ESRF beamline.^[49]^ The fresh electrodes are non‐cycled, whereas the aged electrodes were cycled 75 times from 3 V to 4.4 V in ethylene carbonate/ethyl methyl carbonate (EC : DMC) 1 : 1 (Sigma–Aldrich, LP30) using a 2016 coin cell case (MTI Corp) and a 0.5 mm thick stainless steel spacer. After 75 cycles, the capacity retention of the undoped NMC811 was 71.4 % whereas for Zr‐NMC811 was 83.8 % as reported in Figure S2. Prior to the measurements, the coin cells were opened and the electrodes were stored inside an Argon filled glovebox. For the measurements, the electrodes were first removed from the glovebox, then, a sharp scalpel was used to cut a triangle shaped portion with a thin tip out of the electrode. The triangle portions were fixed on a sample holder and the scans were performed close to their tip. The beam was centred ~0.60 mm below the tip of the triangle shaped cut electrode. The electrodes were exposed to air for 20 min before the measurement scan started. The tomographic scans were carried out using an incident X‐ray beam with an energy of 29.6 keV and a flux of 1.42×10^12^ photons s^−1^. Using the holotomography technique,^[50]^ four scans were performed at different sample‐detector propagation distances. Each scan involved the recording of 2505 projections, using a PCO edge 5.5 camera (2560×2160 pixels^2^). The sample was rotated 360° during the scan, with an exposure time of 15 ms per step. The entire acquisition process took approximately 15 minutes per electrode. 3D reconstructions were achieved in two steps: (i) phase retrieval calculation using an in‐house developed octave script based on a Paganin‐like approach using a delta/beta ratio of 119, and (ii) filtered backprojection reconstruction using ESRF software PyHST2 for a final volume of 64×64×54 μm^3^and a voxel size of 25 nm.

### TEM and SEM

Pristine undoped NMC811 and pristine Zr‐NMC811 particles for TEM measurements were cut using a focused ion beam (FIB, JIB‐4700F, JEOL) in order to obtain thin slices of approximately 70 nm to 100 nm thick. Between undoped NMC811 and Zr‐NMC811, homogenous and similar particles in size were chosen for the FIB cut. Each selected particle was covered by a Platinum (Pt) layer to protect it during the fine milling process at high voltages while using the ion beam. Two spots of each lamella were chosen for selected area (electron) diffraction (SAED) measurement, one close to the surface and the other in the bulk of the secondary particle. The SEM micrographs were taken using Tescan MIRA III scanning electron microscope at accelerating voltage of 20 kV in secondary electron mode.

### X‐Ray Spectroscopy Data Collection

XAS data were collected at the BM23 beamline of ESRF^[51]^ on fresh and aged electrodes for the undoped NMC811 and Zr‐NMC811 samples. The fresh electrodes are non‐cycled, instead the aged electrodes were cycled 75 times from 3 V to 4.4 V in ethylene carbonate/ethyl methyl carbonate (EC : DMC) 1 : 1 (Sigma–Aldrich, LP30) using a 2016 coin cell case. After 75 cycles, the capacity retention of the undoped NMC811 was 71.4 % whereas for Zr‐NMC811 was 83.8 % as reported in Figure S2. Unlike the tomography measurements described in subsection “Phase‐Contrast Tomography (Nano‐CT)”, the electrodes were placed in an Aluminum holder and sealed with Kapton tape inside an Argon filled glovebox. Due to their oxidation state sensitivity, the electrodes remained sealed throughout the entire measurement process and were returned to the glovebox afterward. Ni, Mn and Co K‐edges were measured in transmission mode. The TMs abundance in the material allowed to have sufficient counts, leading to a collection of a maximum of 3 spectra, that were averaged afterwards. Respective metal foils after the sample were used as a reference to calibrate the data collected from the beamline. The x‐ray absorption near edge spectroscopy (XANES) data processing for normalization and reference correction was performed using Athena software.^[52]^

## Conflict of Interests

The authors declare no conflict of interest.

4

## Supporting information

As a service to our authors and readers, this journal provides supporting information supplied by the authors. Such materials are peer reviewed and may be re‐organized for online delivery, but are not copy‐edited or typeset. Technical support issues arising from supporting information (other than missing files) should be addressed to the authors.

Supporting Information

## Data Availability

The data that support the findings of this study are available from the corresponding author upon reasonable request.
