# Supplementary material for: Operando Investigation of Zr Doping in NMC811 Cathode for High Energy Density Lithium Ion Batteries
Source: ChemSusChem. 2025 Jan 21;18(8):e202401796. doi: 10.1002/cssc.202401796 (PMC11997945; doi:10.1002/cssc.202401796)
Supplement: Supplementary file 1 — Supporting Information [file CSSC-18-e202401796-s001.pdf]

ChemSusChem

Supporting Information

Operando Investigation of Zr Doping in NMC811 Cathode for High Energy Density Lithium Ion Batteries

Mattia Colalongo, Basit Ali, Nikita Vostrov, Michal Ronovský, Marta Mirolo, Valentin Vinci, Cesare Atzori, Isaac Martens, Peter Kúš, Andrea Sartori, Lide Yao, Hua Jiang, Tobias Schulli,* Jakub Drnec,* Timo Kankaanpää, and Tanja Kallio*

Supporting Information: Operando Investigation of Zr Doping in NMC811 Cathode for High Energy Density Lithium-ion Batteries

Mattia Colalongo,†,‡ Basit Ali,‡ Nikita Vostrov,† Michal Ronovský,† Marta Mirolo,† Valentin Vinci,† Cesare Atzori,† Isaac Martens,† Peter Kúš,¶ Andrea Sartori,† Lide Yao,§ Hua Jiang,§ Tobias Schulli,*,† Jakub Drnec,*,† Timo Kankaanpää,∥ and Tanja Kallio*,‡

†*European Synchrotron Radiation Facility, 71 Avenue des Martyrs, Grenoble 38000, France*

‡*Department of Chemistry and Material Science, School of Chemical Engineering, Aalto University, Kemistintie 1, Espoo 02150, Finland*

¶*Department of Surface and Plasma Science, Faculty of Mathematics and Physics, Charles University, V Holešovičkách 2, Prague 8 18000, Czech Republic*

§*Nanomicroscopy Center, Aalto University, 02150 Espoo, Finland*

∥*Umicore Battery Materials Finland Oy, 67101 Kokkola, Finland*

E-mail: schulli@esrf.fr; drnec@esrf.fr; tanja.kallio@aalto.fi

S1 Beam Dose Estimation

At ID31 beamline the photon flux prior to the sample is measured by a photodiode. The measured value is also used for instensity normalization as the overall flux slightly varies over time each time a

refill happens in the storage ring. However, the photodiode returns a photocurrent value sensitive to parameters such as, incident beam intensity, beam energy, and silicon intrinsic parameters. The formula used for calculating the photon flux (ϕ) is as follow:

$$\phi = \frac{E_{eh} I}{q_{\mathrm{e}^-} E(1 - e^{-(\mu_{en} t_{Si} \rho_{Si})})} \tag{S1}$$

where E_{eh} is the electron-hole pair energy of the Silicon wafer in the photodiode,[S1] I is the measured photocurrent, q_{e^-} is the coulombic charge of an electron, E the energy of the incident beam, μ_{en} is the mass-energy attenuation coefficient value extrapolated from Figure S1, t_{Si} and ρ_{Si} are thickness and density of the Silicon photodiode respectively. Once the photon flux is calculated, the beam dose estimation on the cathode materials can be retrieved by using the Jupyter Notebook tool at `https://github.com/STardif/dose_estimation`.

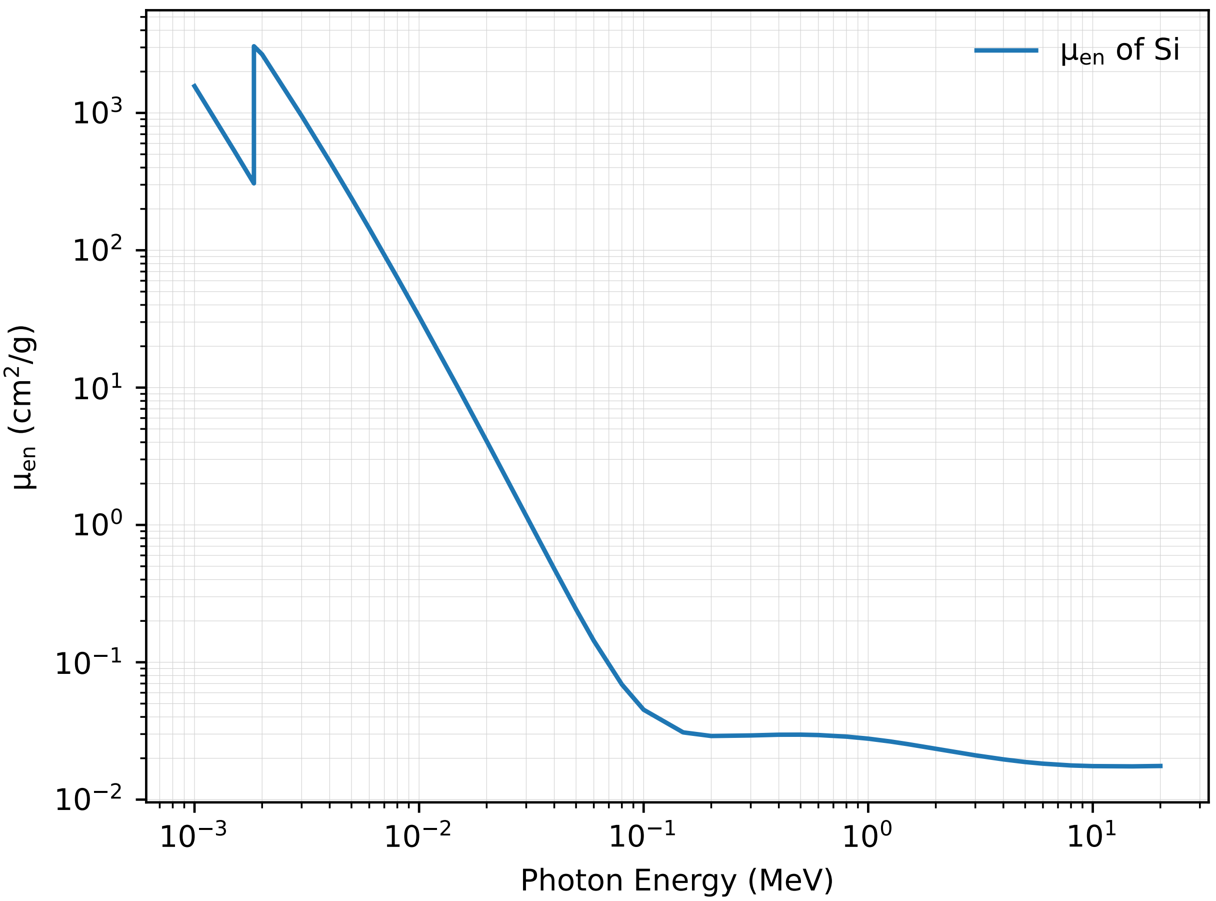

Figure S1: μ_{en} mass-energy attenuation coefficient of Silicon as function of the incident beam energy

S2 Electrochemistry

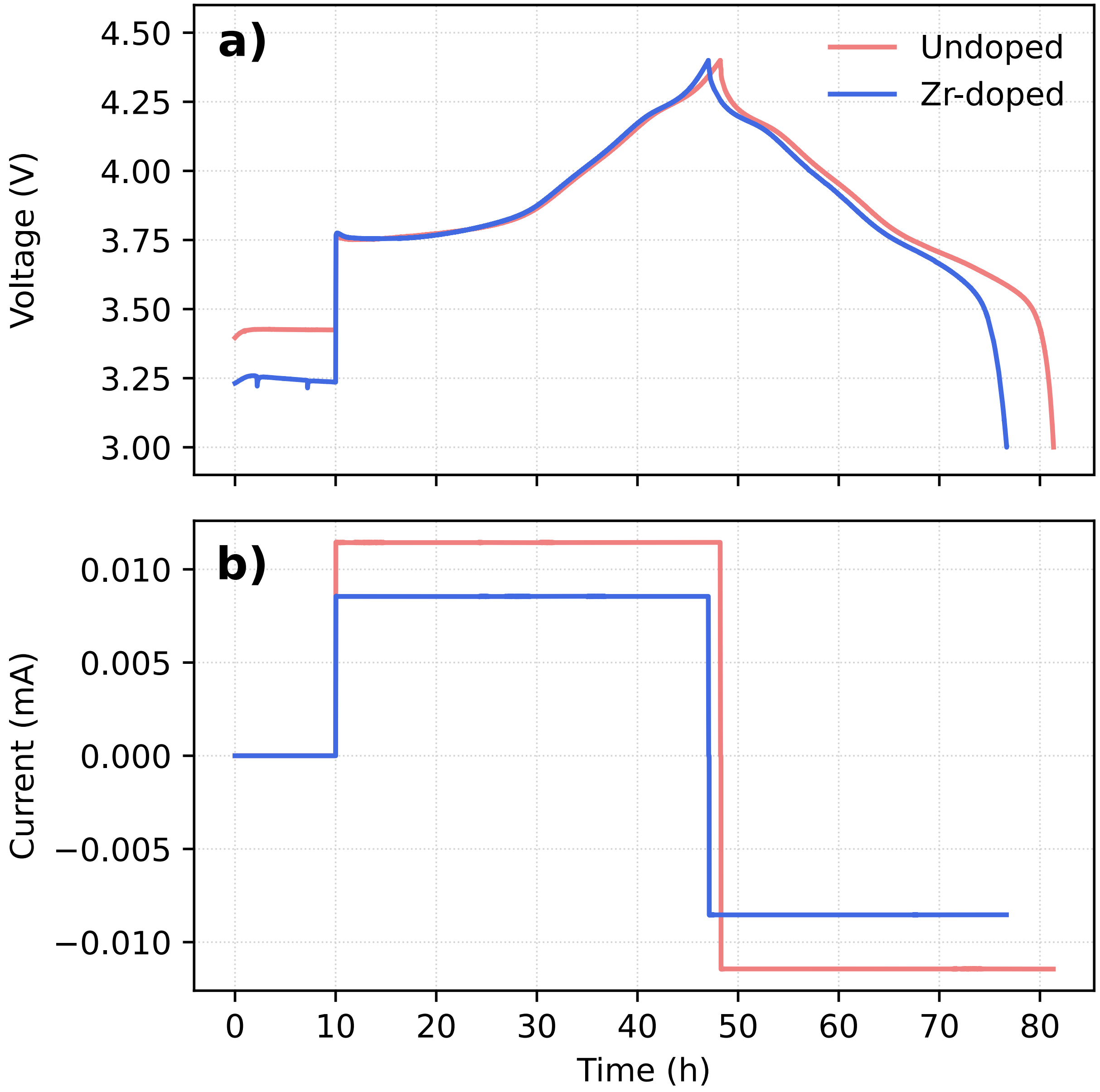

Figure S2: Formation cycle for undoped and Zr-doped cells at ∼0.025C: **a)** shows voltage variation over time. Charge and discharge process run for $\sim 80\,\mathrm{h}$, which is an expected time for the applied c-rate. **b)** shows applied current over time for both samples. Current varies due to their slightly different active masses.

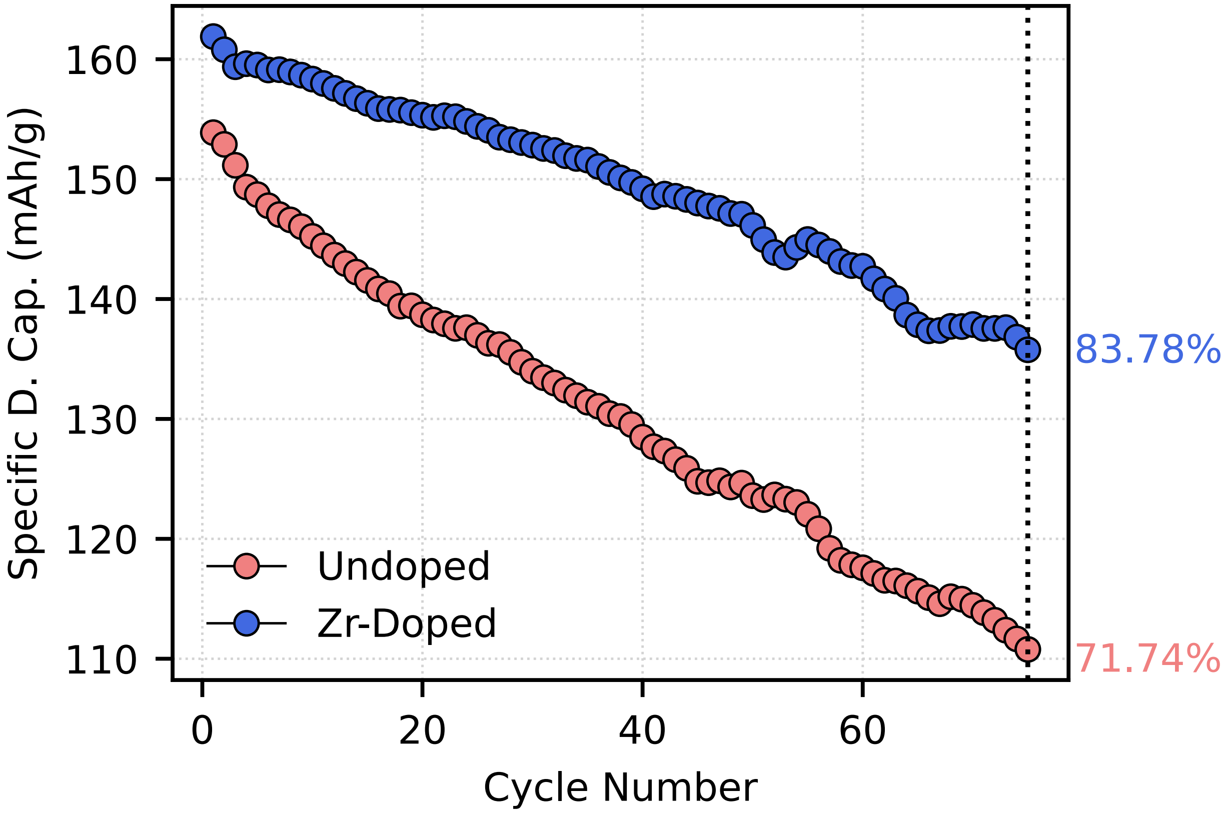

Figure S3: undoped NMC811 and Zr-NMC811 samples cycled 75 times. The cells were continuously checked and once a consistent degradation difference was reached (∼20%), cells were stopped.

S3 Diffraction

Peak amplitude, position and width are individually extracted for each diffractogram by using the *lmfit* python module. However, due to the presence of multiple peaks an auto-detection condition in the python script is used. The script uses two pseudo-voigt functions when i) the peak intensity drops to a certain threshold and ii) whether through the 2nd derivative of the diffraction peak, multiple local minima are observed. The detection of local minima are therefore used as initial peak position for the fit, ensuring a reliable starting guess point. As illustrated in Figure S6, when a second peak appears, the script is capable to pinpoint their center through the f''(2θ) local minima analysis which is clear to be an initial valid guess.

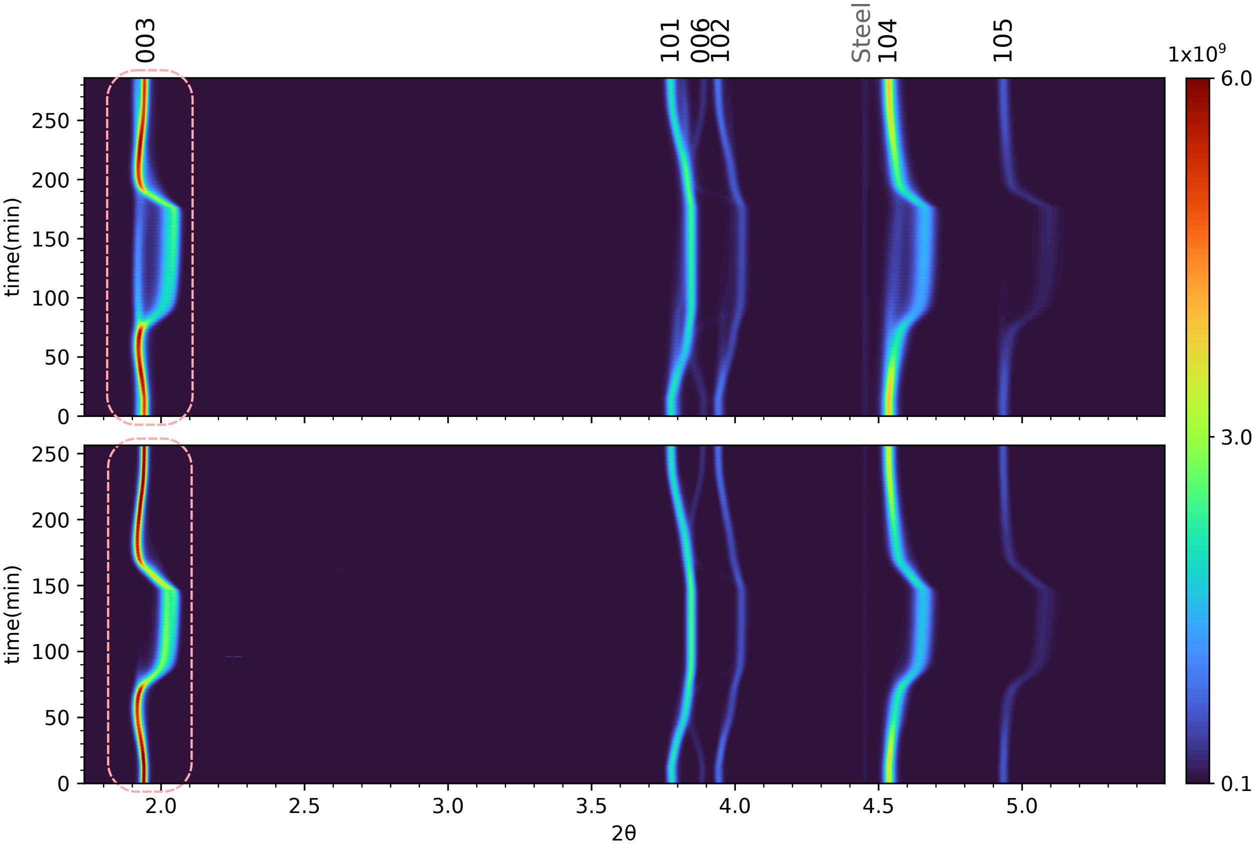

Figure S4: In this image is reported a broader 2θ range for undoped NMC811 (top panel) and Zr-NMC811 (bottom panel). The circled area represent the 003 XRD reflection selected to study the c-axis evolution of the NMC811 sample. Between $4.4°$ and $4.5°$ a constant reflection is observed in both samples. The low intensity peak is assigned to the 111 austenite reflection coming likely from the stainless steel parts of the Swagelok cell

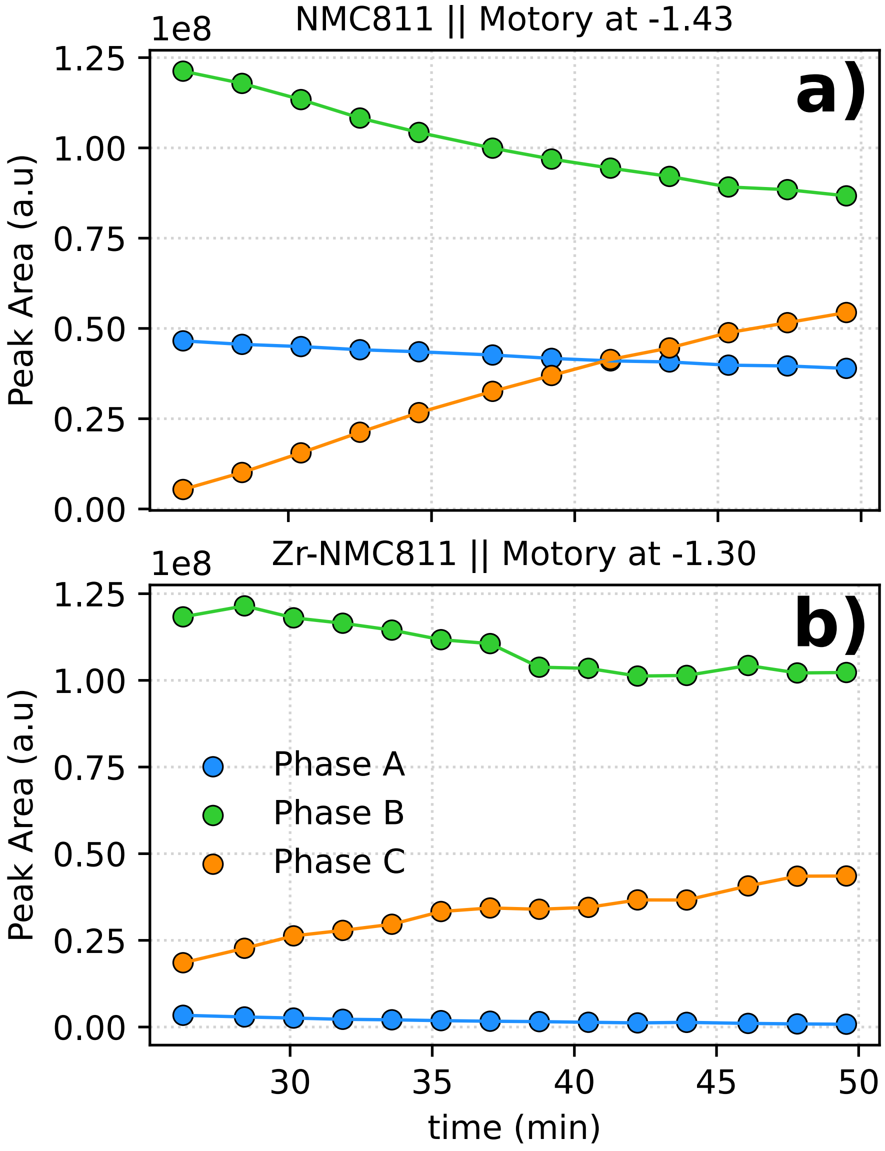

Figure S5: a) Amplitude variation of phase A, B and C for undoped NMC811, fixing the motor position to Y=-1.43. **b)** Amplitude variation of phase A, B and C for Zr-NMC811, fixing the motor position to Y=-1.3. By considering only one Y position, for both samples, the amplitude sharp variations disappeared.

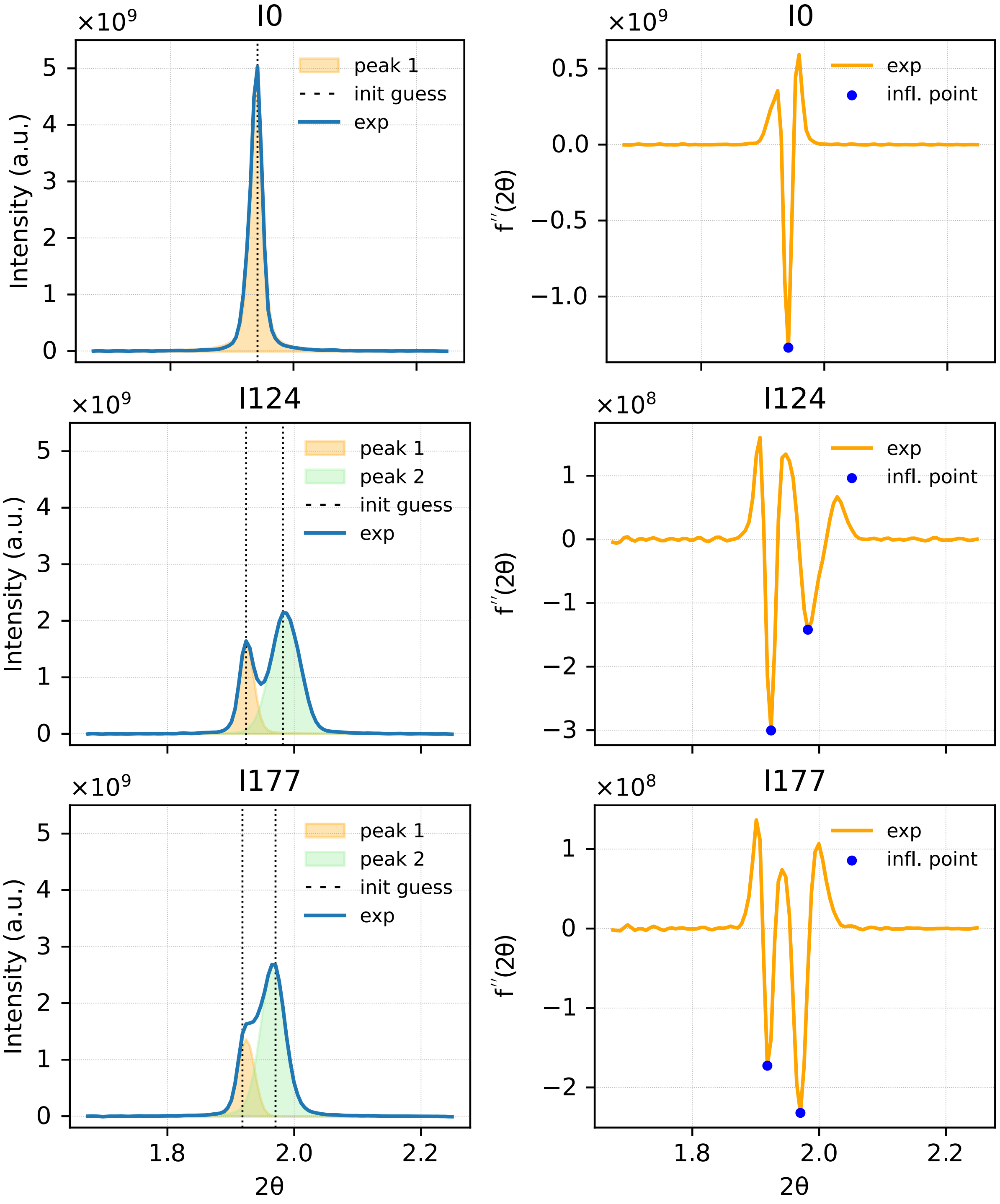

Figure S6: Automatic peak fitting of diffraction data: On the left column diffraction peaks at different state of charge (SOC). On the right column the corresponding f''(2θ).

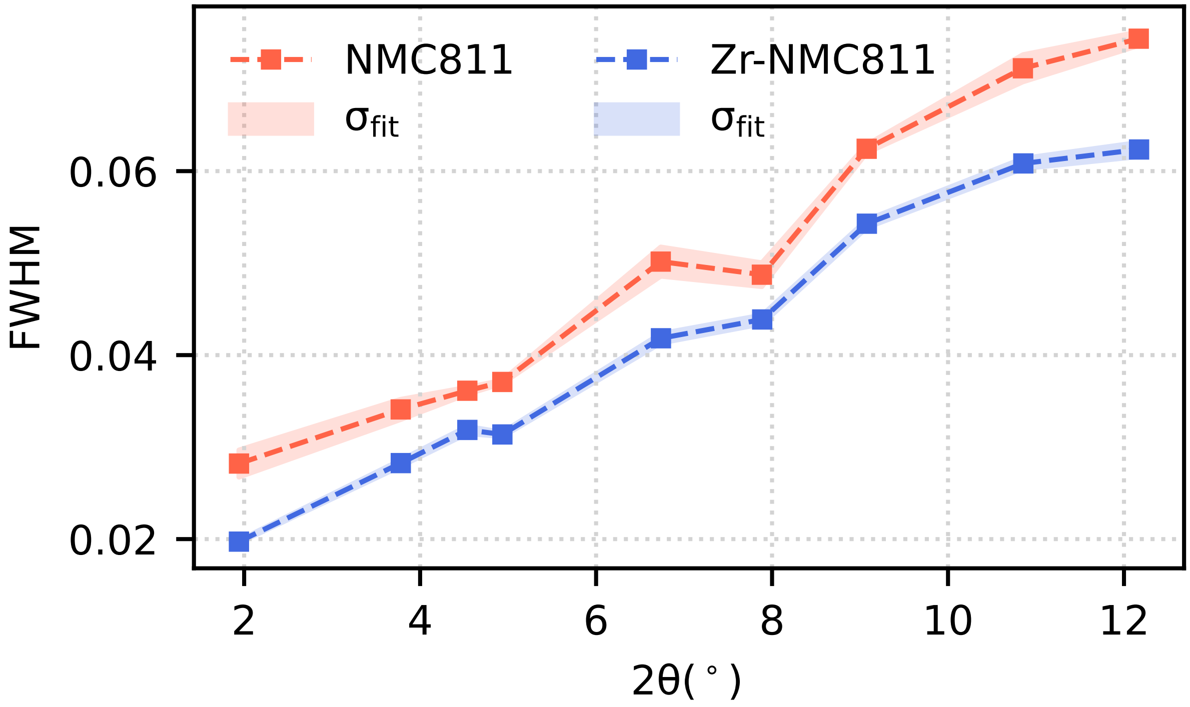

Figure S7: Full Width Half Maximum (FWHM) fit of undoped NMC811 and Zr-NMC811 samples over 9 peaks. The FWHM extraction is needed to extract the μ-strain of the sample through the Williamson-Hall plot. The fill-in between each scatter point represents the standard deviation (σ) of the fitting by means of *lmfit* python module

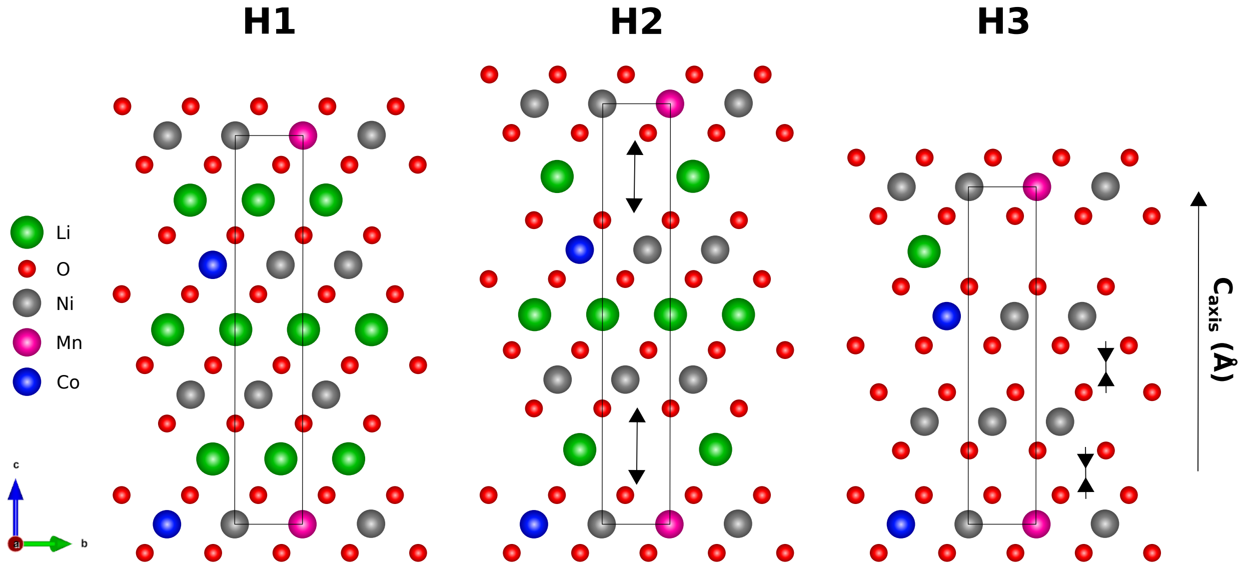

Figure S8: NMC811 phase transformation. H1 is the initial hexagonal phase fully lithiated. The hexagonal H2 phase is achieved upon lithium extraction from the cathode material. When Li^+ is extracted from the H1 structure, the oxygens in the Li-slab start to repel each other. The H2 therefore expands along the c-axis of the unit cell because of the oxygens electrostatic repulsion. At high charge voltages, Li^+ extracted is so high that the oxygen electrostatic repulsion is not sufficient to keep the structure stable. Hence, the cell shrinks and collapses. This phase is reported in the literature as hexagonal H3.

S4 X-ray Absorption Spectroscopy

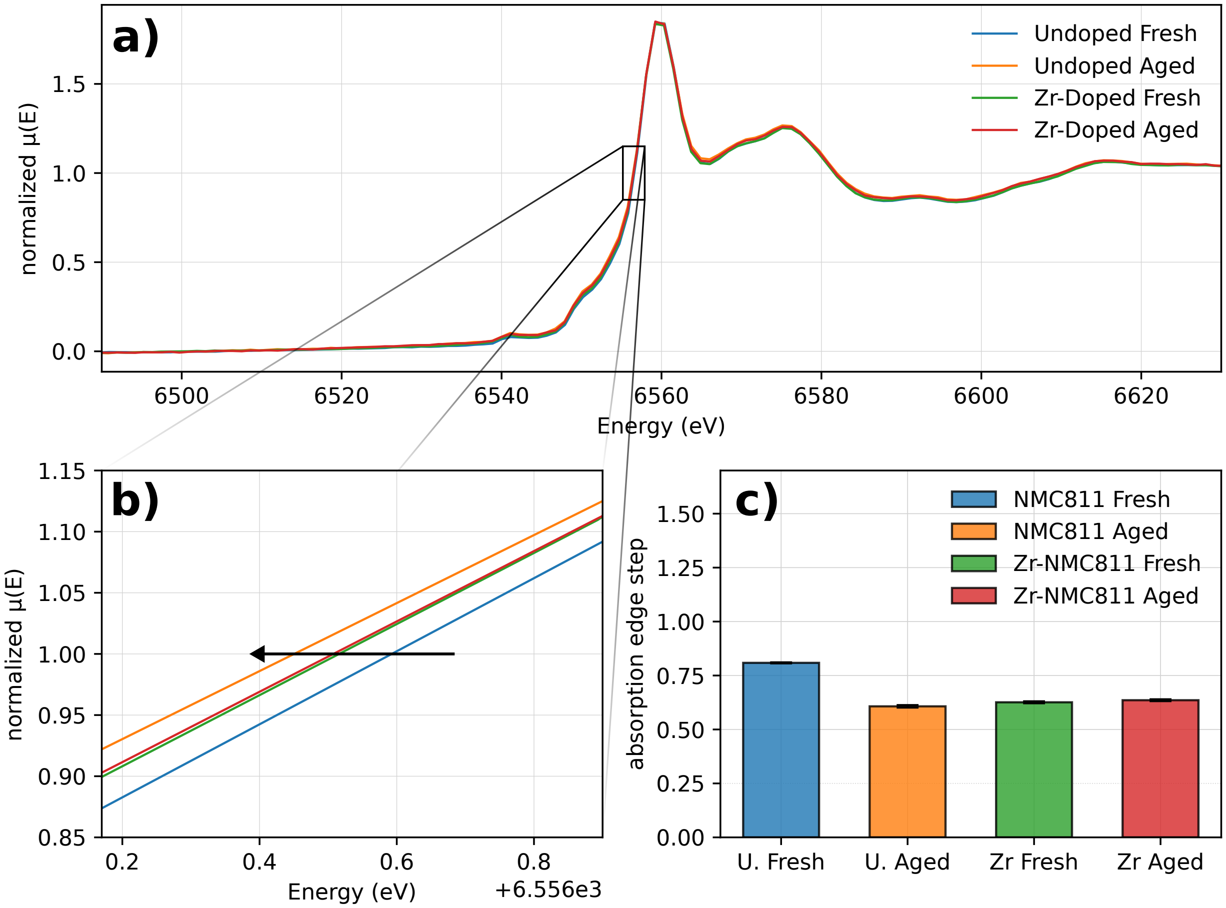

Figure S9: Mn k-edge of fresh and aged undoped NMC811 and Zr-NMC811 samples. In **a)** XANES portion of the Mn k-edge. In **b)** a magnified region close to E_0 that shows the energy shift clearer. **c)** Extracted edge-step values from Athena software. Error bars are barely visible but are calculated by considering the standard deviation over 4 scans values

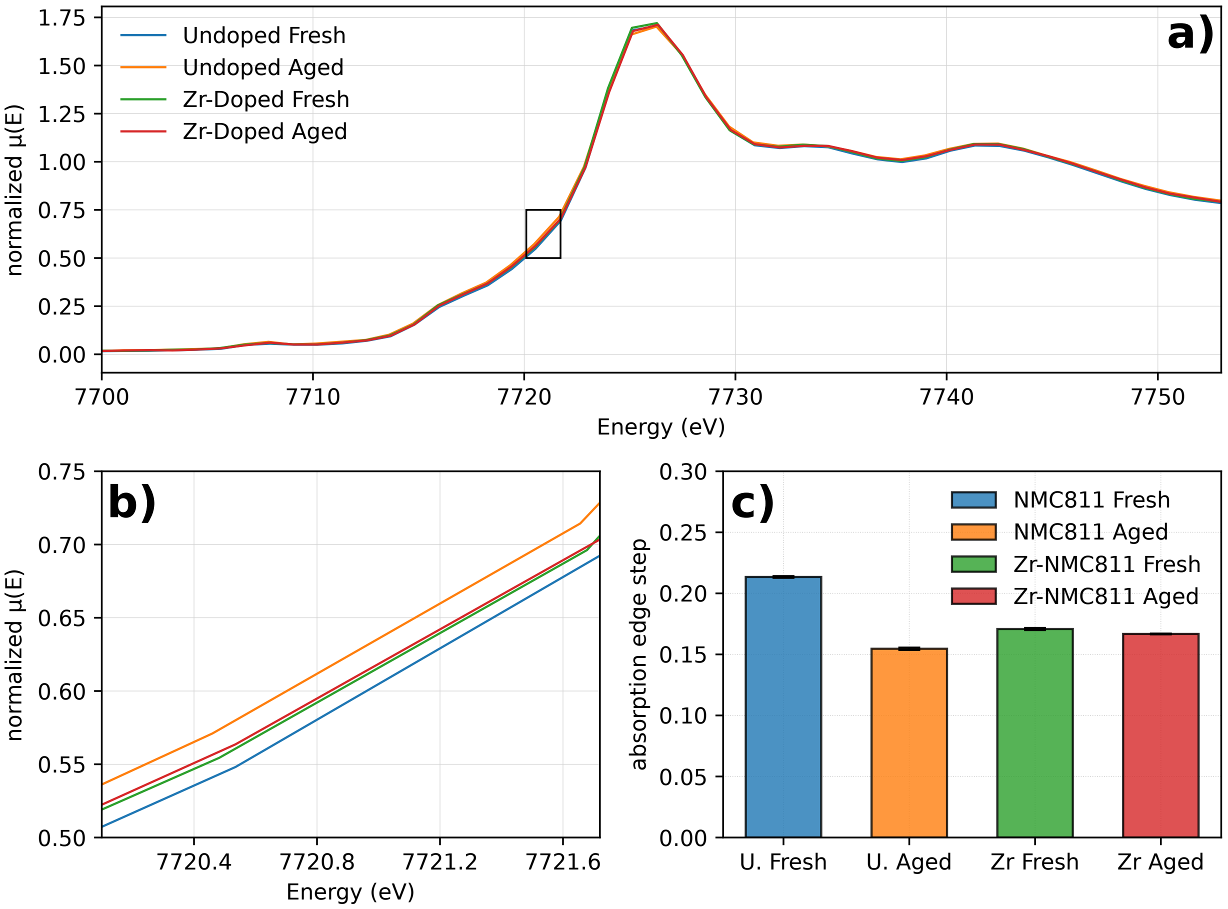

Figure S10: Co k-edge of fresh and aged undoped NMC811 and Zr-NMC811 samples. In **a)** XANES portion of the Co k-edge. In **b)** a magnified region close to E_0 that shows the energy shift clearer. **c)** Extracted edge-step values from Athena software. Error bars are barely visible but are calculated by considering the standard deviation over 4 scans values

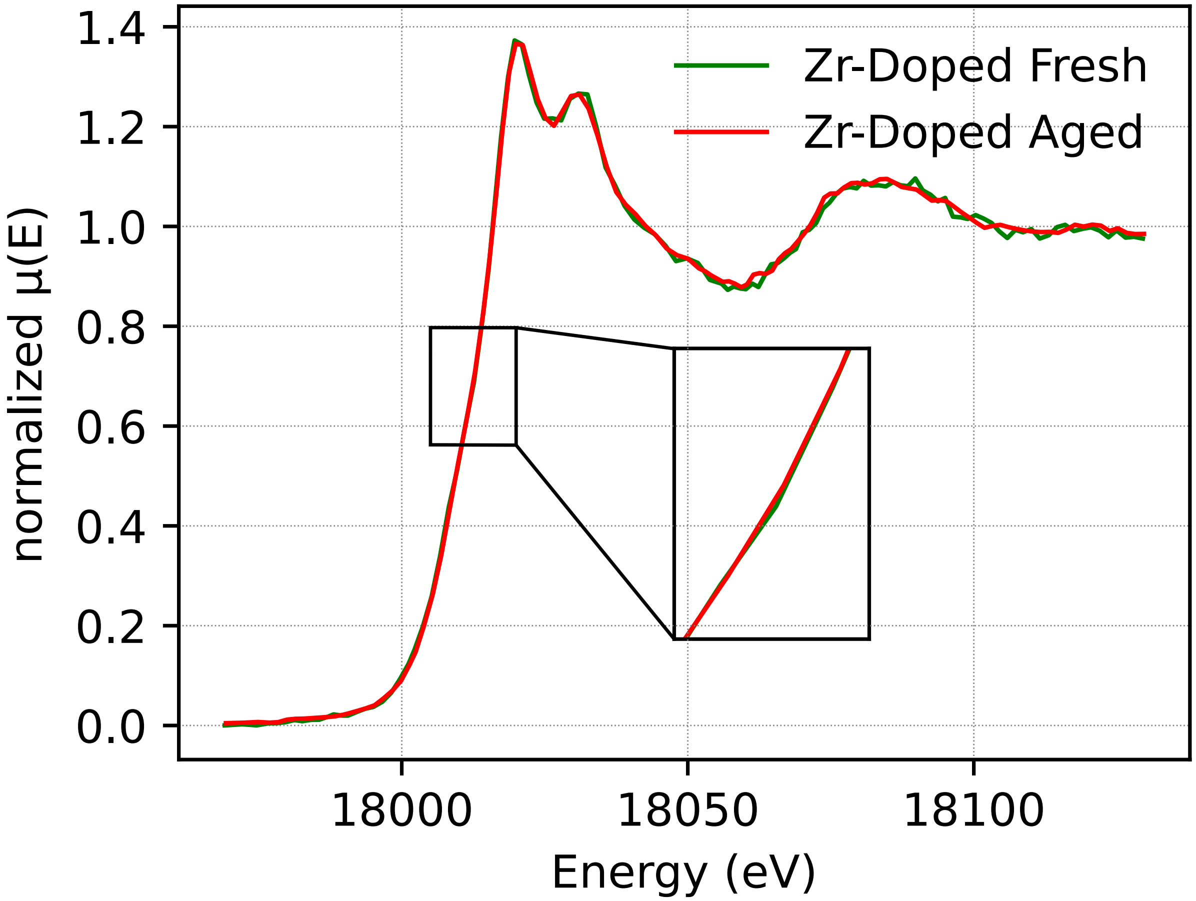

Figure S11: Zr k-edge of fresh and aged Zr-NMC811 sample. It is reported in the XANES portion of the Zr K-edge spectrum. Magnified region close to E_0, which shows no clear peak shift.

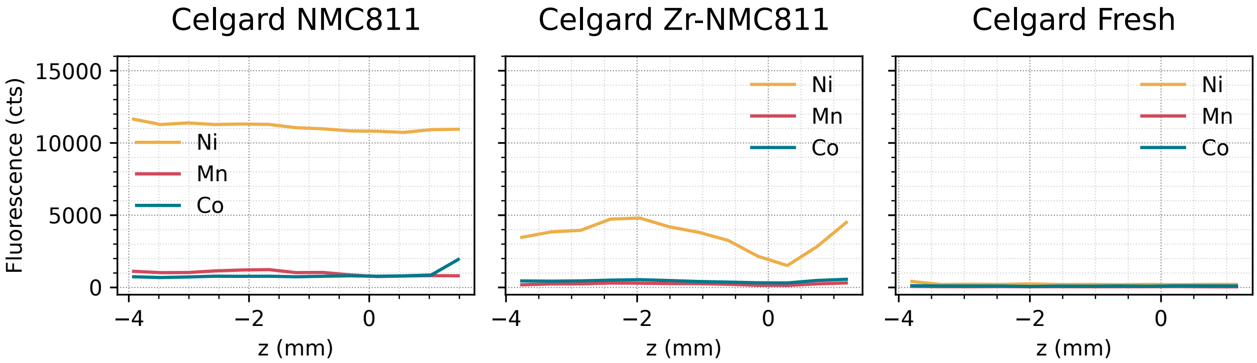

Figure S12: Fluorescence maps of aged Celgard membranes for undoped NMC811, Zr-NMC811 and fresh samples. The beam was firstly set to the membrane center and a scan along z-axis was performed.

S5 Phase Contrast X-Ray Nano-tomography

To evaluate the porosity of each particle, the slices that compose the particle were threshold in intensity to segment the particle of interest (brighter pixels). A series of regions of interest (ROI's) were drawn at initial, middle and final part of the sliced particle using the *Wand tool* in Fiji software.[S2] In this way we can approximate with the *Interpolate ROI's* function the particle area for the multiple slices. An example of the procedure is reported in Figure S13 where the particle segmentation is defined by the yellow contour line. Once the segmentation of the particle is performed, sequential measurement on each slice is run. The outcome parameter we focus on is the *Area Fraction*, which is defined as the percentage of red pixels within the selected area. Therefore, voids in the particle not seen as red/bright pixels will decrease the values of Area percentage due to porosity in the particle slice. In conclusion, the higher is the porosity inside a particle the lower the *Area Fraction* values will be.

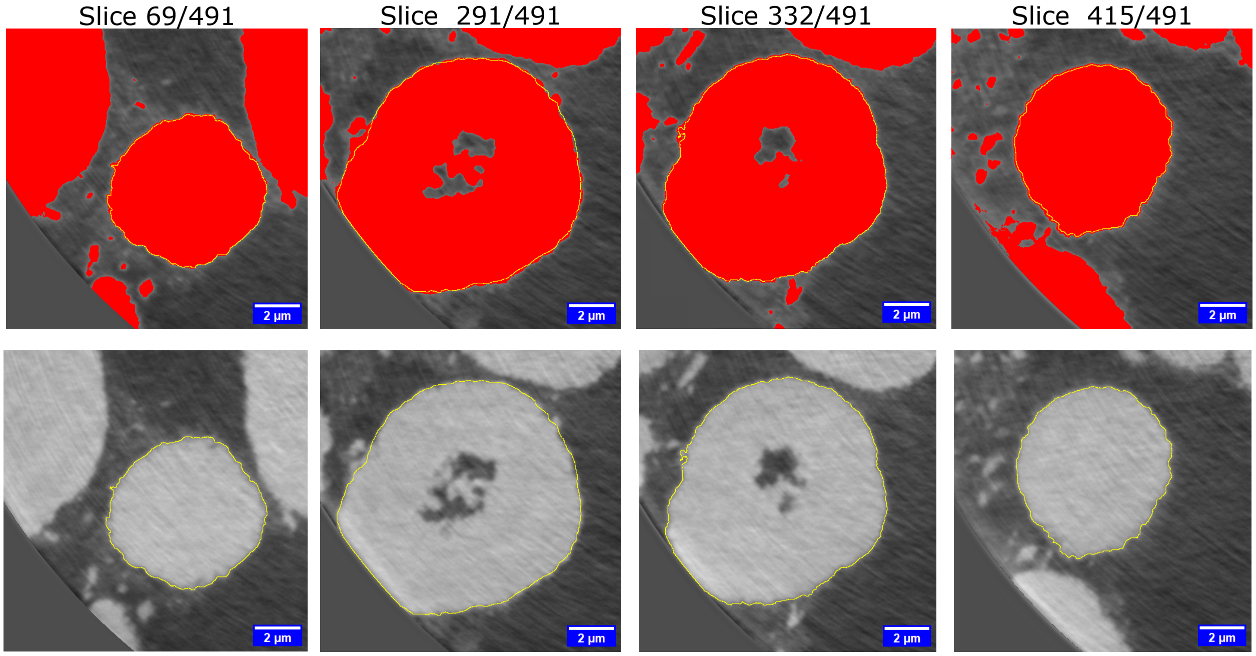

Figure S13: Example of ROI selection among different slices using the Wand and interpolation tools given by Fiji software. In the **Top row** the image is intensity threshold to effectively select the area of the particle and remove the surrounding. The **Bottom row** shows the ROI selection through thresholding (yellow contour line) onto the original particle

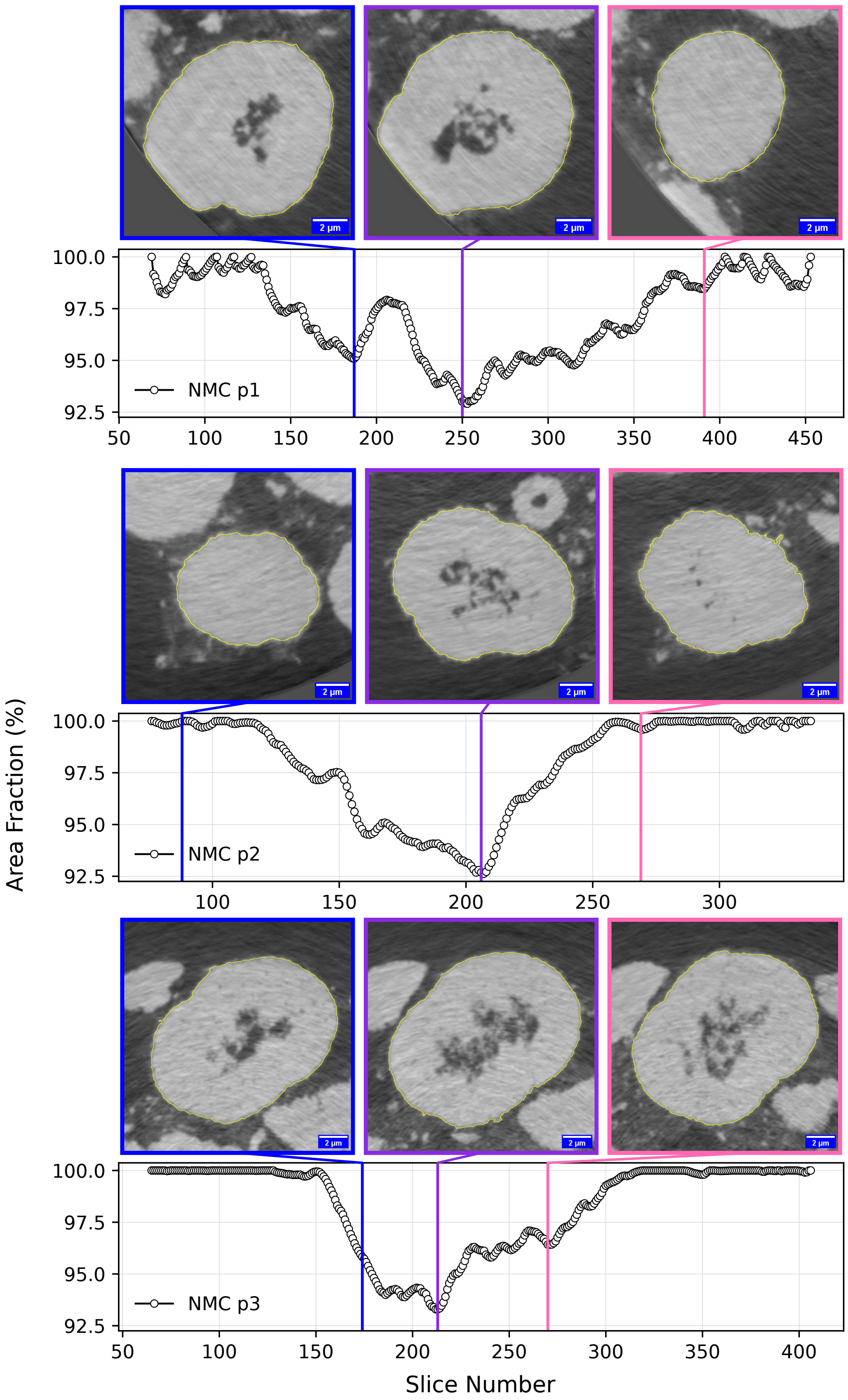

Figure S14: The image shows the *Area Fraction* per each slice of 3 different NMC particles. To differentiate possible local and maximum porosity minima, 3 different slices are visualized in Blue, Purple and Magenta

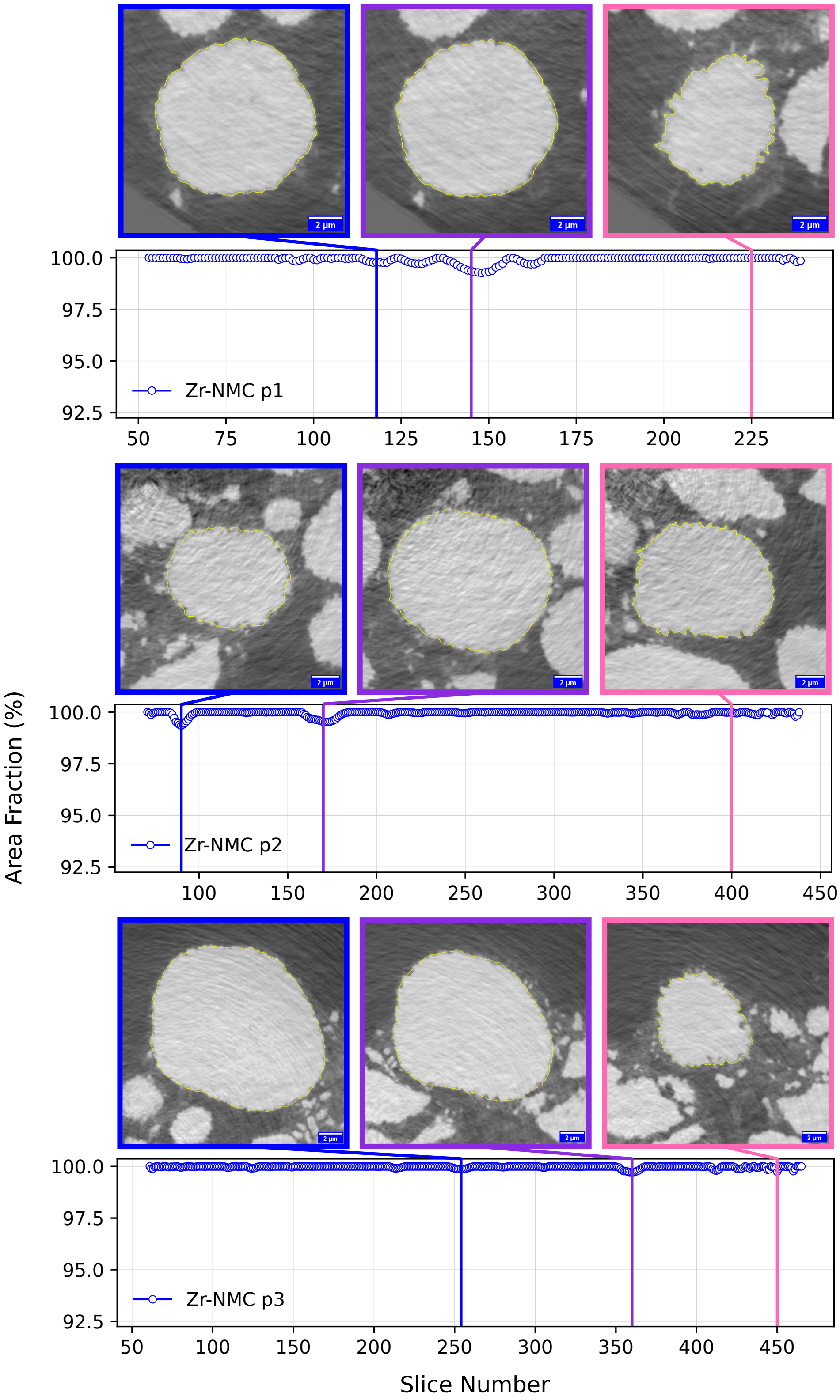

Figure S15: The image shows the *Area Fraction* per each slice of 3 different Zr-NMC particles. To differentiate possible local and maximum porosity minima, 3 different slices are visualized in Blue, Purple and Magenta

References

(S1) Scholze, F.; Rabus, H.; Ulm, G. Mean energy required to produce an electron-hole pair in silicon for photons of energies between 50 and 1500 eV. *Journal of Applied Physics* **1998**, *84*, 2926–2939.

(S2) Schindelin, J.; Arganda-Carreras, I.; Frise, E.; Kaynig, V.; Longair, M.; Pietzsch, T.; Preibisch, S.; Rueden, C.; Saalfeld, S.; Schmid, B.; others Fiji: an open-source platform for biological-image analysis. *Nature methods* **2012**, *9*, 676–682.
